# A conserved node degree-based backbone and flexible hub organization of brain connectome during naturalistic movie watching

**DOI:** 10.1016/j.neuroimage.2026.122171

**Published:** 2026-08-20

**Authors:** Xuehu Wei, Laura Rigolo, Colin P Galvin, Einat Liebenthal, Yanmei Tie

**Affiliations:** a Department of Neurosurgery, Brigham and Women’s Hospital, Boston, Massachusetts, USA; b McLean Imaging Center, McLean Hospital, Belmont, Massachusetts, USA; c Harvard Medical School, Boston, Massachusetts, USA

**Keywords:** Naturalistic stimuli, Movie-watching fMRI, Functional connectome, Inter-subject functional correlation, Degree-based backbone, Rich-club organization, Stimulus-brain association

## Abstract

How does the brain organize and transmit information during naturalistic conditions? Using large-scale movie-watching fMRI data spanning 14 diverse clips, we revealed a degree-based dual architecture of the functional connectome under naturalistic stimuli. During movie watching, the brain consistently expresses a conserved network backbone characterized by persistently high node degree in temporal and occipital sensory cortices and parietal association regions, whereas anterior higher-order regions show relatively lower node degree and substantially greater variability across different clips. We further found that this backbone links brain network organizational patterns to stimulus features of the clips, particularly audiovisual features capturing human presence and social communication. Building on this conserved backbone, different movie clips recruit distinct sets of rich-club hubs, reflecting a flexible hub organization distributed across superior temporal gyrus, temporo-parieto-occipital junction, precuneus/posterior cingulate cortex, intraparietal sulcus, and visual motion-sensitive regions. These hubs serve as key integrative nodes whose connectivity statistically mediates the relationship between stimulus features and cross-network integration. The conserved backbone and rich-club organization were reproduced in an independent movie-watching dataset analyzed with an identical pipeline, providing partial validation of these topological findings. Together, these findings reveal a network-level organizing principle in which a conserved backbone supports stable large-scale coordination, while flexible hub organization enables feature-specific coupling between stimulus features and distributed brain networks.

## 1. Introduction

Nervous systems operate as networks that transmit and process information (Laughlin and Sejnowski, 2003; Seguin et al., 2023), with communication and co-activation across brain regions forming the connectome that underlies cognitive processing (Lachaux et al., 2001; Salinas and Sejnowski, 2001). During real-life experiences, the brain coordinates activity across distributed regions to process complex information, supporting two core functions: transforming sensory inputs into representations of the external world (Goodale and Milner, 1992; Hickok and Poeppel, 2007), and integrating these inputs with internal states, such as intentions, motivations, and predictions, to construct coherent perceptual interpretations (Friston, 2010). Such integration is thought to rely on coordinated interactions within large-scale brain networks, yet how these interactions are organized under naturalistic conditions remains incompletely understood.

Movie watching has emerged as a widely adopted naturalistic paradigm for studying brain function (Hasson et al., 2004; Rajimehr et al., 2024; Yang et al., 2023). Movies are considered naturalistic stimuli because they integrate diverse visual elements, sounds, speech, music, social interactions, and narrative structure, evoking robust and reliable brain activity representative of natural cognition (Finn and Bandettini, 2021; Sonkusare et al., 2019). Consistent with this view, movie stimuli elicit large-scale neural responses across occipital, temporal, and parietal cortices, inferior frontal and orbital prefrontal regions, as well as subcortical structures (Hasson et al., 2010, 2004; Yang et al., 2023). These regions support hierarchical sensory information processing (Hasson et al., 2004; Wilf et al., 2017), language and narrative comprehension (Tie et al., 2015), social and emotional memory functions (Adolphs, 2009; Underwood et al., 2021), and context integration and sustained attention (Vossel et al., 2014), highlighting the brain’s capacity to coordinate multiple specialized systems during diverse, real-world perception.

Despite the engagement of shared large-scale networks, neural responses to movies are not identical across stimuli. Variations in dialogue, narrative structure, and social content shape both the strength and spatial extent of cross-subject neural synchronization (Hasson et al., 2010; Leipold et al., 2024; Li et al., 2025). Movies with rich audiovisual content and coherent narratives evoke widespread synchronization extending into higher-order association regions such as the inferior parietal lobe and posterior cingulate/precuneus (Golland et al., 2007; Hasson et al., 2004), whereas stimuli lacking specific modalities or narrative coherence elicit responses in fewer regions (Hasson et al., 2008). Together, these findings indicate that stimulus content strongly shapes neural responses. However, most prior work has emphasized response magnitude and regional engagement, leaving open whether diverse naturalistic stimuli are supported by a shared large-scale network framework, and which network mechanisms enable content-dependent information transfer across distributed brain systems.

Addressing these questions requires moving beyond region-wise response strength to a network-level description of how distributed interactions are organized. Network neuroscience provides a framework for addressing this gap by characterizing brain function in terms of graph-theoretical properties such as node degree and hub regions (Bassett and Sporns, 2017; Bullmore and Sporns, 2009). Among these properties, rich-club hubs, i.e., highly connected regions that form a central core for global information communication, are thought to support the integration of information across functional systems (Crossley et al., 2013; van den Heuvel and Sporns, 2011). While rich-club organization is well established in resting-state and task-based networks (Power et al., 2010), recent work has begun to extend this graph-theoretical framework to naturalistic paradigms (Jang et al., 2017; Ortiz-Rios et al., 2021), asking whether hub organization also supports information integration during real-world perception.

Prior work therefore suggests that movie content shapes large-scale neural responses and that graph-theoretical approaches can characterize hub organization during naturalistic cognition. However, it remains unclear whether diverse movie stimuli share a conserved degree-based network scaffold, whether rich-club hub organization is flexibly reconfigured across movie contexts, and how such network topological variation relates to multimodal stimulus features. In this study, we addressed these questions by analyzing movie-watching functional magnetic resonance imaging (fMRI) data from the Human Connectome Project (HCP), complemented by an independent in-house dataset. The combination of high image quality, ample sample size, and stimulus heterogeneity provides rich variability and a solid empirical foundation for characterizing the general and specific topological features of brain network organization in response to different movie clips. We constructed stimulus-locked functional networks using inter-subject functional correlation (ISFC) (Rosenthal et al., 2017; Simony et al., 2016) and applied data-driven graph-theoretical modeling (Kim et al., 2018) to characterize large-scale organizational regularities as well as clip-specific reconfigurations of network topology. Specifically, we first tested whether node-degree topology is conserved across diverse movie clips and whether this conserved organization can be *characterized* as a degree-based backbone. Here, we use the term “backbone” to refer to a reproducible degree-centrality topography during naturalistic movie watching. We characterized this backbone using a composite *backbone score* that combined high mean degree, low cross-clip variability, and high rank stability, thereby capturing a reproducible topography of regions that consistently occupied central positions across diverse movie clips. We then examined whether clip-level variation in this degree-based topology covaried with multidimensional stimulus features. We further identified rich-club hubs and investigated whether the recruitment and connectivity patterns of these hubs vary systematically with stimulus content demands (Bolton et al., 2020; Levakov et al., 2023; Ye et al., 2023). Finally, we examined whether stimulus features, rich-club connectivity, and distributed cortical networks showed structured indirect statistical associations.

## 2. Methods

### 2.1. Data

#### 2.1.1. Subjects and experimental paradigm of the Human Connectome Project 7T dataset

The Human Connectome Project (HCP) Young Adult 7T release dataset (https://www.humanconnectome.org/study/hcp-young-adult) was used in this study from the original 184 subjects, data from 176 (106 females and 70 males) subjects who underwent all four movie-watching runs (Movies 1–4) were selected for this study. All subjects were healthy young adults aged between 22 and 35 years, and all scanning sessions were conducted at the Center for Magnetic Resonance Research at the University of Minnesota. For this HCP dataset, each movie run consisted of 4 or 5 clips separated by 20 s of rest and ended with the same validation clip blocks. Movie clips were presented in a fixed order across participants according to the HCP movie-watching protocol, with 20-s rest periods separating adjacent clips. Movie 1 and Movie 3 featured clips from independent films (both fiction and documentary films). The other two runs, Movie 2 and Movie 4, included clips from Hollywood films. For a brief description of each clip, see Supplementary Table 1. All movie clips were sourced from the Human Connectome Project (HCP; https://db.humanconnectome.org/). The detailed MRI acquisition parameters are reported in the Supplementary Methods. All primary analyses focused on the 14 main movie clips, excluding validation clips and inter-clip rest periods.

#### 2.1.2. Subjects and experimental paradigm of the in-house dataset

We used the movie-watching fMRI data that was reported previously (Tie et al., 2015) for this study. This dataset included movie-watching fMRI data from 22 right-handed healthy subjects (11 females and 11 males, mean age = 26.3 years, range: 19–39 years) who had no history of neurological, cognitive, or psychiatric disorders, nor any speech, hearing, or vision deficits. All subjects were native English speakers. The study protocol was approved by the Mass General Brigham Institutional Review Board, and all subjects provided written informed consent. For this dataset, the movie stimulus was a 7-minute excerpt from the family film *The Parent Trap* (1998; directed by Nancy Meyers; produced by The Meyers/Shyer Company and Walt Disney Pictures). The detailed MRI acquisition parameters are reported in the Supplementary Methods.

### 2.2. Data preprocessing

The HCP movie-watching fMRI data were minimally preprocessed using the minimal HCP pipeline of fMRIVolume (Glasser et al., 2013; T Vu et al., 2017). The first 10-sec data of each fMRI clip was discarded to allow for stabilization of the BOLD signal. Preprocessing included gradient distortion correction, TOPUP-based spin echo field map unwarping, motion correction, high-pass filtering with a cutoff of 2000 s, brain-boundary-based registration of EPI to structural T1 images, non-linear registration to Montreal Neurological Institute (MNI) standard space and grand-mean intensity normalization. fMRI data were cleaned up for head motion, cardiac pulsation, breathing, and scanner artifacts using sICA+FIX denoising. In addition, the average whole-brain signal at each TR was calculated using an HCP-provided per-subject, per-run mask (brainmask_fs.1.60.nii.gz) and regressed out from the FIX-denoised images. The data were then spatially smoothed using a 4 mm Full Width at Half Maximum (FWHM) Gaussian kernel and interpolated to 2 mm isotropic voxels.

To account for the hemodynamic delay, we included 5 TRs (5 s) following the ending of each clip in the functional connectivity calculation, and the remaining 15 TRs (15 s) of the 20-second rest period between clips were excluded from the analysis.

The in-house dataset movie-watching fMRI data were preprocessed using the pipeline developed by the CBIG group (https://github.com/ThomasYeoLab/CBIG/tree/master/stable_projects/preprocessing/CBIG_fMRI_Preproc2016). The first 10-sec data of fMRI was discarded. Then, motion correction was performed using spline interpolation, and Framewise displacement (FD) and DVARS were calculated. Frames with excessive motion (FD > 0.2) or signal changes (DVARS > 50) were regarded as outliers. The corrected EPI images were registered to individual structural T1-weighted images and subsequently normalized to the 2 mm MNI152 standard space. fMRI data were denoised by regressing out several confounding signals, including the global mean signal, white matter (WM) and cerebrospinal fluid (CSF) signals, as well as 12 motion-related regressors (6 original parameters and their 6 temporal derivatives). Band-pass filtering (0.01–0.1 Hz) was then applied using a regression approach to capture neural signals relevant to both rest and task states, followed by spatial smoothing with a 4-mm full-width at half maximum (FWHM) Gaussian kernel.

### 2.3. Network analysis and statistics

**Fig. 1.**
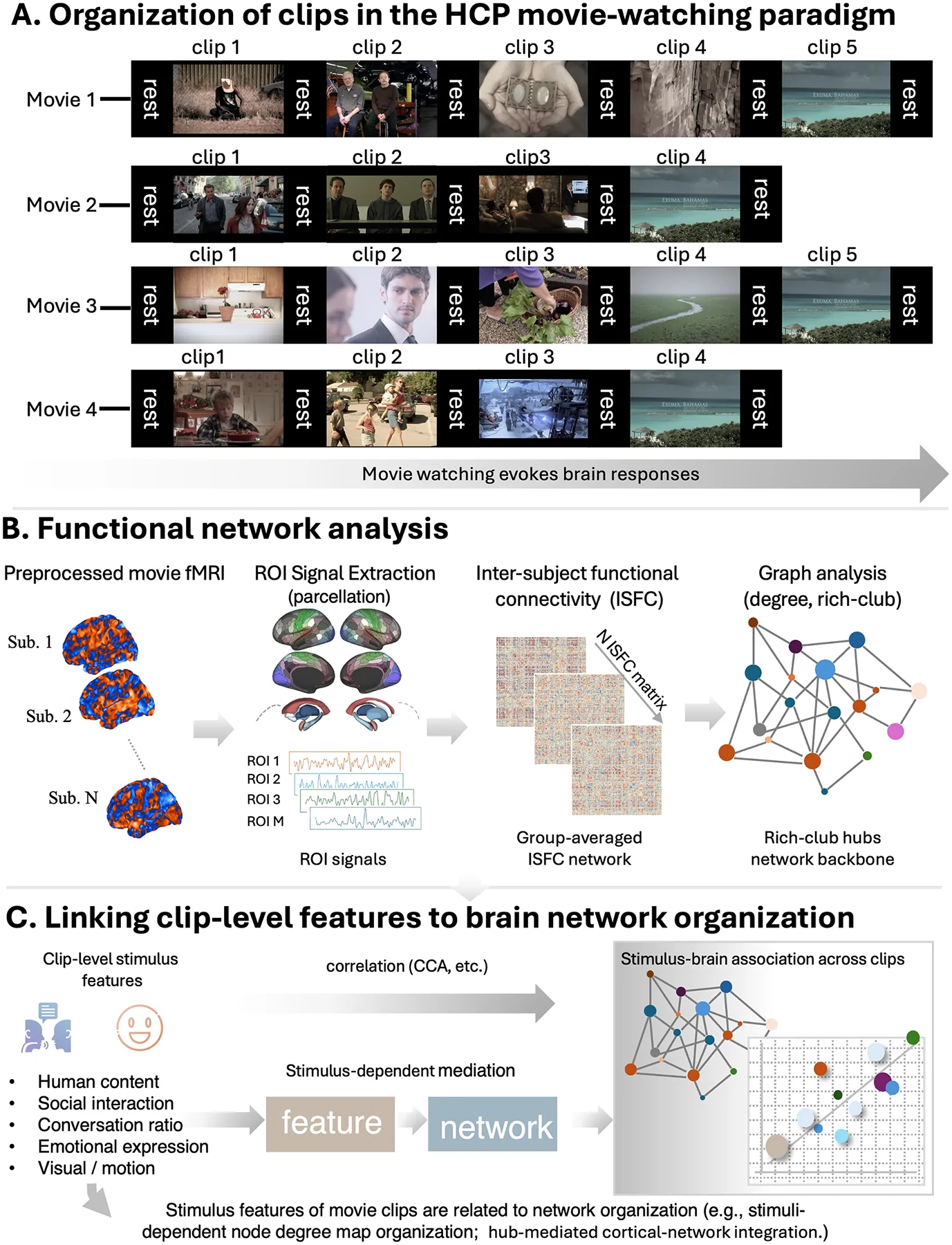
Movie-watching fMRI stimuli and data analysis workflow. (A) Organization of movie clips in the Human Connectome Project (HCP) movie-watching paradigm. Participants underwent four movie fMRI runs composed of multiple clips (about·1–4 min) presented in a fixed order and interleaved with brief rest periods (20 s). (B) Functional network analysis workflow. Preprocessed fMRI data were parcellated into cortical regions-of-interest (ROIs), and inter-subject functional connectivity (ISFC) was computed for each movie clip to capture stimulus-locked functional synchronization across individuals. Group-averaged ISFC networks were then analyzed using graph-theoretical measures, including node degree and rich-club organization, to identify integrative hub regions forming a core network backbone during movie watching. (C) Linking clip-level features to brain network organization. Clip-level visual semantic, auditory and social (e.g., face number, social interaction strength, and visual motion) were related to large-scale network measures using multivariate analyses. Feature–network mediation analyses further assessed how rich-club hub–to–network connectivity supports feature-specific processing within each cortical network.

The overall analysis workflow is illustrated in Fig. 1B. Briefly, fMRI time series were extracted from 374 regions of interest. For each movie clip, we computed group-averaged inter-subject functional correlation (ISFC) matrices (Simony et al., 2016) to estimate stimulus-locked functional connectivity. Functional networks were constructed by retaining the strongest 30% of connections, with sensitivity analyses repeated across 10%–60% proportional density thresholds. These networks were used to quantify global graph properties, including global efficiency and modularity, as well as regional node-degree topology. Node-degree maps were further used to define a conserved degree-based backbone, and rich-club analyses were performed to identify hub organization. Finally, we related clip-level network variation to multi-modal stimulus features.

### 2.4. Brain network construction

For each HCP movie clip and for the in-house dataset, the brain functional network was constructed on 374 regions of interest (ROIs) including 360 cortical ROIs (180 in each hemisphere) defined by the HCP MMP 1.0 atlas (Glasser et al., 2016) and 14 subcortical ROIs (7 in each hemisphere) defined by the Harvard-Oxford atlas (https://fsl.fmrib.ox.ac.uk/fsl/docs/#/other/datasets). Preprocessed voxel-wise fMRI signals were averaged within each ROI to generate regional time series, which was subsequently z-transformed across time points to normalize signal amplitude. For each subject, the weighted brain network connections were constructed via a leave-one-out inter-subject functional correlation (ISFC) approach (Simony et al., 2016). ISFC is a powerful method for studying brain function during task states, offering improved signal-to-noise ratio (SNR) and a more comprehensive view of functional integration compared to traditional intra-subject network analyses (Kim et al., 2018; Simony et al., 2016). For each subject, the ISFC matrix represented the functional connectivity of each ROI calculated by the Pearson correlation between the time course of that subject and the average time course of all other ROIs across all other subjects. Correlation coefficients were Fisher z-transformed and averaged across subjects to obtain a group-level ISFC matrix for each clip. The ISFC connectivity matrix was thresholded using a one-sample t-test: p < 0.01 (Bonferroni correction) for the HCP dataset and p < 0.01 (FDR correction) for the in-house dataset. Significant group-level ISFC matrices were then used for network construction and graph-theoretical analyses.

### 2.5. Graph metrics calculation

We used graph-theoretical network measures to characterize the topology of the functional connectome (Bullmore and Bassett, 2011) in this study. For a given network matrix, each brain ROI was defined as a node, and the connectivity between ROIs as an edge. Then the graph metrics of each mean-ISFC of each clip was computed using the Brain Connectivity Toolbox for Python (https://brainconn.readthedocs.io/). At the node level, degree was computed to quantify regional connectivity strength. At the global level, we assessed network efficiency and modularity to characterize large-scale integration and segregation. Node-degree maps served as the primary regional representation for backbone characterization and multivariate stimulus–brain analysis. We further quantified rich-club organization (van den Heuvel and Sporns, 2011), a property of complex networks in which a subset of highly connected nodes (hubs) are more densely interconnected and support efficient global communication. Statistical significance was assessed by comparison with degree-preserving randomized networks (1000 permutations), yielding normalized rich-club coefficients. Rich-club regions were defined as nodes with degree exceeding the threshold that maximized the normalized rich-club coefficient for each clip. Across clips, we further assessed the centrality profile of rich-club regions using participation coefficients (Rubinov and Sporns, 2010) to distinguish connector versus provincial hubs. Detailed definitions, formulas, and statistical procedures for graph metrics and rich-club analysis are provided in the Supplementary Methods. The same graph-metric calculation procedures, including node definition, degree calculation, proportional thresholding at the primary 30% network density, and rich-club analysis, were applied to both the HCP and in-house datasets.

### 2.6. Cross-clip stability of node-degree topology

To evaluate whether node-degree topology was conserved across movie clips, we computed pairwise Pearson correlations between the 14 clip-wise node-degree maps and summarized cross-clip stability as the mean pairwise correlation. This analysis was performed at the primary 30% network density threshold and repeated across proportional density thresholds from 10% to 60% to assess threshold sensitivity. To assess robustness, we performed a whole-pipeline subject-level bootstrap analysis with 5000 iterations. In each iteration, participants were resampled with replacement, clip-wise group-averaged ISFC networks were reconstructed using the same edge-level significance and thresholding procedure, node-degree maps were recalculated, and mean cross-clip similarity was recomputed. We further tested the significance of the observed similarity using ROI-label permutation null models with 5000 permutations. In the unconstrained null model, ROI labels were randomly shuffled across the whole brain for each clip. To reduce the possibility that cross-clip similarity was driven only by coarse anatomical organization, we additionally implemented an anatomical-group-restricted null model. Anatomical group assignments were derived from the atlas-level grouping of each ROI in the HCP-MMP parcellation (Glasser et al., 2016), yielding 22 cortical anatomical groups per hemisphere, with subcortical ROIs treated as an additional separate group. In each anatomical-group-restricted permutation, ROI labels were shuffled only within the same anatomical group for each clip, preserving broad lobe-level spatial organization while disrupting region-specific correspondence across clips. For each null iteration, mean cross-clip degree-map similarity was recomputed and compared with the observed value.

### 2.7. Definition of the degree-based backbone

Building on the clip-wise node-degree maps derived from the group-averaged ISFC network of each movie clip, we operationalized the degree-centrality backbone using three complementary cross-clip metrics for each ROI: mean degree, coefficient of variation (CV), and rank stability across the 14 clips. Mean degree indexed average regional integration across clips, CV quantified cross-clip variability, and rank stability was defined as the fraction of clips in which an ROI ranked within the top quartile of node degree. These measures were computed at the primary 30% network density threshold and repeated across 10%–60% densities for sensitivity analyses. We then computed a continuous backbone score for each ROI as z(mean degree) − z(CV) + z(rank stability), with each measure z-scored across ROIs before combination. The resulting backbone-score map was used to represent the spatial topography of the degree-based backbone across movie clips. High-backbone-score regions therefore represent data-driven regions with higher average degree, lower cross-clip variability, and greater rank stability across movie clips. To localize regions most strongly expressing this topography, we additionally identified core backbone regions using explicit criteria: top-quartile mean degree, below-median CV, and rank stability of at least 0.75 across clips.

To assess the stability of the degree-based backbone, we performed whole-pipeline resampling and permutation analyses, including subject-level bootstrap resampling (1000 iterations), split-half reliability analysis (1000 random splits), clip-level generalization analyses, and ROI-label permutation null tests (5000 permutations). In all resampling analyses, the complete ISFC-to-degree pipeline was recomputed for each clip before recalculating ROI-wise backbone metrics, rather than resampling precomputed backbone scores. Reliability and generalization were summarized using bootstrap confidence intervals, split-half map similarity, exhaustive clip-partition analyses, leave-one-movie-out correlations, and unconstrained or anatomical-group-restricted ROI-label permutation tests, with full details provided in the Supplementary Methods.

### 2.8. Cortical-level and functionally based framework for rich-club network categorization

To interpret rich-club core locations and enable subsequent system-level mediation analyses, we summarized rich-club organization at the level of 22 cortical regions and one subcortical group, following the ROI to cortical-area grouping annotations provided by the HCP-MMP1.0 atlas (Glasser et al., 2016). The full names and abbreviations of each cortical area are provided in Supplementary Table 4. For each clip, ROI-level rich-club hubs were first identified, and hub membership was then aggregated within each cortical area to characterize cross-clip patterns of hub recruitment at a coarse anatomical scale.

To further characterize the functional architecture of rich-club regions, we divided the whole brain into four large-scale functional groups based on anatomical and functional affiliations (Glasser et al., 2016): speech, visual, motor, and higher-order domain-general systems, and rich-club regions were assigned to their respective functional group. For each movie clip, we computed the ISFC patterns of rich-club regions within each subgroup relative to the whole brain. Specifically, we extracted the ISFC profile of each rich-club region and visualized its connectivity pattern to the rest of the brain regions, highlighting differences across functional subgroups. This approach enabled us to investigate how rich-club regions, distributed across distinct functional systems, dynamically reorganized their connectivity in response to different stimulus conditions.

### 2.9. Stimulus feature extraction and clip-level feature representation

To quantify stimulus features, we leveraged the TR-resolved semantic annotations provided with the HCP 7T movie-watching dataset (https://balsa.wustl.edu/). These annotations label the presence of multiple stimulus features at each time point for every clip using WordNet-based categories, which were summarized for visualization using word clouds (Supplementary Fig. 8). The resulting feature set captures a set of predefined visual semantic dimensions spanning human and social signals as well as scene and object content, including: Human, Body, Face, Building/indoor/outdoor, Road and traffic environment, Transportation, Natural environment/landscape, Objects/props/food, Location/shop, Displacement/motion, Posture/stillness, Visual perception/gaze, Communication action, Emotional expression, and Object manipulation (Supplementary Fig. 8). In addition to these visual annotations, we extracted speech- and social-related features at both TR and clip levels. Speech presence was detected using pyannote (Plaquet and Bredin, 2023), and face-related features were extracted using Pliers (McNamara et al., 2017). To quantify human presence, we detected people in each frame using YOLO (Redmon and Farhadi, 2018) and computed the number of individuals per TR. We further defined a measure of social strength as a composite index of social intensity for each clip (Martynenko et al., 2025).

For each clip, TR-level features were summarized into clip-level measures, including speech occupancy, conversation ratio, people count, and a social-strength index combining close-range human presence, interaction-related semantic cues, and communication-related annotations. Detailed feature definitions, person-detection thresholds, and interaction-cue labels are provided in the Supplementary Methods. The resulting clip-level feature matrix contained 14 clips × 19 features capturing clip-level stimulus feature profiles (see Fig. 4A and Supplementary Fig. 9) and was used for subsequent dimensionality reduction and brain–stimulus association analyses.

### 2.10. Multivariate stimulus-brain canonical correlation analysis

A multivariate stimulus-brain canonical correlation analysis (CCA) (Wang et al., 2020) between clip-level node degree maps and clip-level stimulus feature profiles was performed to test whether clip variations in node degree organization covary with stimulus features. Node-degree maps from thresholded group-level ISFC networks were arranged into a 14 × 374 clip-by-ROI brain matrix, and the stimulus features formed a 14 × 19 clip-by-feature matrix. To reduce dimensionality and limit overfitting given the small number of clips, principal component analysis (PCA) was applied separately to the brain and stimulus matrices, and the first two PCs from each matrix were retained for CCA. The corresponding PCA loading maps for the brain and stimulus features are shown in Supplementary Fig. 10.

Statistical significance was assessed using a full-pipeline permutation test in which clip labels were randomly shuffled between the brain and stimulus matrices. For each permutation, the full analysis was repeated, including standardization, PCA dimensionality reduction, CCA fitting, and computation of the canonical correlation. This procedure was repeated 10,000 times to generate a null distribution for the observed canonical correlation. The same pipeline was also repeated across 10%–60% proportional network density thresholds to assess threshold sensitivity. To interpret the canonical axes, we computed structure coefficients by correlating each original ROI degree value or stimulus feature with its corresponding canonical score across clips. These coefficients were used to visualize the low-dimensional stimulus–brain axis and to identify stimulus features significantly associated with the canonical mode after FDR correction. Out-of-sample stability was evaluated using leave-one-clip-out cross-validation and compared against a full-pipeline permutation null.

### 2.11. Exploratory indirect-association analysis of rich-club hub pathways

To explore whether movie stimulus features were statistically associated with large-scale network integration statistically linked by rich-club hubs connectivity, we performed a mediation-inspired exploratory path analysis framework. Following the notation of statistical mediation frameworks (Imai et al., 2010), we used the (a), (b), and (ab) terms to summarize indirect associations, but these estimates were interpreted descriptively and were not taken as evidence for causal or mechanistic mediation. For each mediation model, the input (X) was each clip-level stimulus feature, the intermediate connectivity measure (M) was defined as the mean functional connectivity between a given rich-club hub and all ROIs within a target cortical region group and the outcome (Y) was defined as within-target-network connectivity, computed as the mean functional connectivity among all ROI pairs within that target region group. Target cortical region groups were defined using ROI-to-region grouping annotations from the HCP-MMP1.0 atlas (Glasser et al., 2016). (M) and (Y) were estimated separately for each participant and clip from subject-level adjacency matrices derived from the preprocessed ISFC networks at the primary 30% network density threshold. Analyses were restricted to rich-club regions identified as rich-club members in at least six clips.

For each rich-club hub × target-network pathway, path coefficients were fitted within each participant across the 14 clips. Specifically, path a quantified the association between the clip-level stimulus feature and hub-to-target connectivity (X→M), and path b quantified the association between hub-to-target connectivity and within-target-network connectivity while accounting for the stimulus feature (M→Y). The participant-level indirect-association term was computed as (ab), and these participant-level estimates were then summarized across participants. This procedure avoided treating participant-by-clip observations as independent pooled samples. When a rich-club hub belonged to the target division, the seed hub was excluded from both the (M) and (Y) estimates.

Significance and uncertainty were assessed at both participant and clip levels. Participant-level bootstrap resampling was used to estimate the group-level mean indirect term and its 95% confidence interval (5000 iterations). Statistical significance was assessed using clip-label permutation testing (5000 permutations), in which the clip labels of the stimulus feature were shuffled while preserving participant-specific connectivity profiles, yielding permutation-based p values (p_perm_) and standardized z statistics (z_perm_). Pathway-level significance was corrected using Benjamini–Hochberg FDR correction across both rich-club hub and target-network margins, and pathways surviving both margins were considered significant (q < 0.05). Because the stimulus feature (X) varied only across the 14 clips, we additionally computed delete-one-clip jackknife confidence intervals to quantify uncertainty related to stimulus sampling.

## 3. Results

**Fig. 2.**
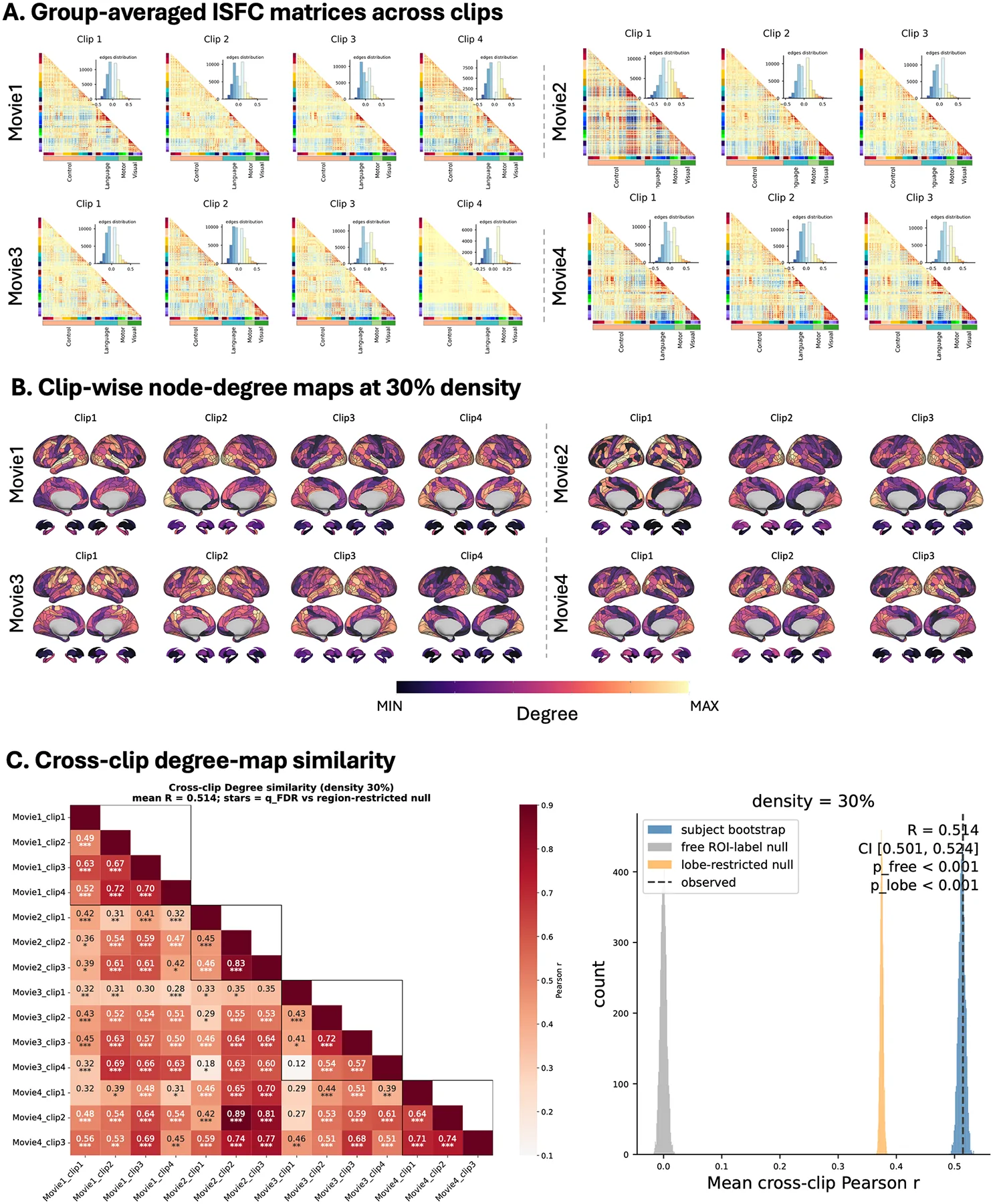
Clip-wise ISFC networks and degree-map stability at the primary 30% network density threshold. (A) Group-averaged ISFC matrices for each movie clip. (B) Clip-wise node-degree maps computed from thresholded group-averaged ISFC networks. All maps are displayed using the same color scale from blue to red, indicating negative to positive ISFC values. (C) Cross-clip similarity of node-degree maps. Left: pairwise similarity matrix across the 14 clips. Right: whole-pipeline subject-level bootstrap and null analysis of mean cross-clip degree-map similarity. The observed similarity exceeded both the unconstrained ROI-label null and the anatomical-group-restricted null distributions.

We first constructed clip-wise ISFC networks to characterize stimulus-locked functional connectome organization during naturalistic movie watching. Group-averaged ISFC matrices showed distinct but broadly structured connectivity patterns across movie clips (Fig. 2A). We next examined whether the regional node-degree topology derived from these networks was conserved across clips.

### 3.1. Robust global network properties across density thresholds justify a common analysis regime

We began at the global level by evaluating key graph properties of the whole-brain functional connectome derived from group-averaged ISFC across a range of proportional density thresholds, constructed by retaining the strongest connections at each density level, focusing on global efficiency and modularity. While all movie clips exhibited highly similar trends across density thresholds from 10% to 60%, the absolute values of these metrics varied across clips at each threshold (Supplementary Fig. 1; Supplementary Tables 2–3), consistent with differences in stimulus content. Global efficiency (upper panel of Supplementary Fig. 1), which indexes the capacity for large-scale functional integration during movie watching, showed clip-dependent variability, with maximum differences of 0.04, 0.07, 0.08, and 0.02 for movie runs 1–4, respectively. Modularity (lower panel of Supplementary Fig. 1), reflecting the extent to which the functional connectome is organized into segregated communities, also varied across clips, with differences of 0.10, 0.12, 0.20, and 0.08 observed for movie runs 1–4, respectively. The global network metrics showed consistent threshold-dependent trends across movie clips and became relatively stable above approximately 30% density (Supplementary Fig. 1). We therefore retained 30% density as the primary threshold for visualization and main analyses, while treating threshold robustness as a separate sensitivity analysis. Threshold-sensitivity results for the main findings are reported in the relevant sections below and in the Supplementary Materials.

### 3.2. Conserved degree-centrality topography across movie clips

To examine whether the degree-based backbone reflected a reproducible degree-centrality topography during naturalistic movie watching, we first tested whether regional node-degree patterns were conserved across clips. For each of the 14 primary clips, we quantified the node degree of each ROI (total 374 ROIs), i.e., the number of connections between an ROI and the rest of the brain that represents large-scale functional integration (Bullmore and Sporns, 2009). Fig. 2B shows the node-degree map for each clip at the primary 30% density threshold. To quantify cross-clip stability, we computed the mean pairwise Pearson correlation among the 14 clip-wise node-degree maps and evaluated its reliability using whole-pipeline subject-level bootstrap resampling and permutation-based null models (whole-brain ROI-label shuffling, and a stricter shuffling restricted to within region groups that preserves local spatial autocorrelation; see methods). At 30% density, the observed mean cross-clip similarity was moderate (R = 0.514; Fig. 2D). Whole-pipeline subject-level bootstrap resampling yielded a highly consistent estimate of this similarity (bootstrap 95% CI = [0.501, 0.524]). The observed similarity exceeded both the unconstrained ROI-label null distribution (mean R = −0.0001, permutation p < 0.001) and the anatomical-group-restricted null distribution (mean R = 0.375, 97.5th percentile = 0.380, permutation p < 0.001), indicating that the observed correspondence could not be explained solely by broad anatomical regional organization.

At the pairwise level, 85 of the 91 clip pairs showed significant node-degree map similarity after FDR correction (p_FDR < 0.05). Similarity was generally stronger within movie runs (Movie 1: R = 0.49–0.72; Movie 2: R = 0.45–0.83; Movie 3: R = 0.12–0.72; Movie 4: R = 0.64–0.74). Descriptively, cross-run similarity was higher among Hollywood clips (Movies 2 and 4; mean R = 0.670) than among independent clips (Movies 1 and 3; mean R = 0.481). This pattern was consistent across density thresholds from 10% to 60%, with full threshold-sensitivity results reported in Supplementary Figs. 2 and 3. Together, these analyses provide positive evidence for a conserved degree-centrality topography across clips.

### 3.3. Node degree-based backbone is conserved across diverse movie clips

**Fig. 3.**
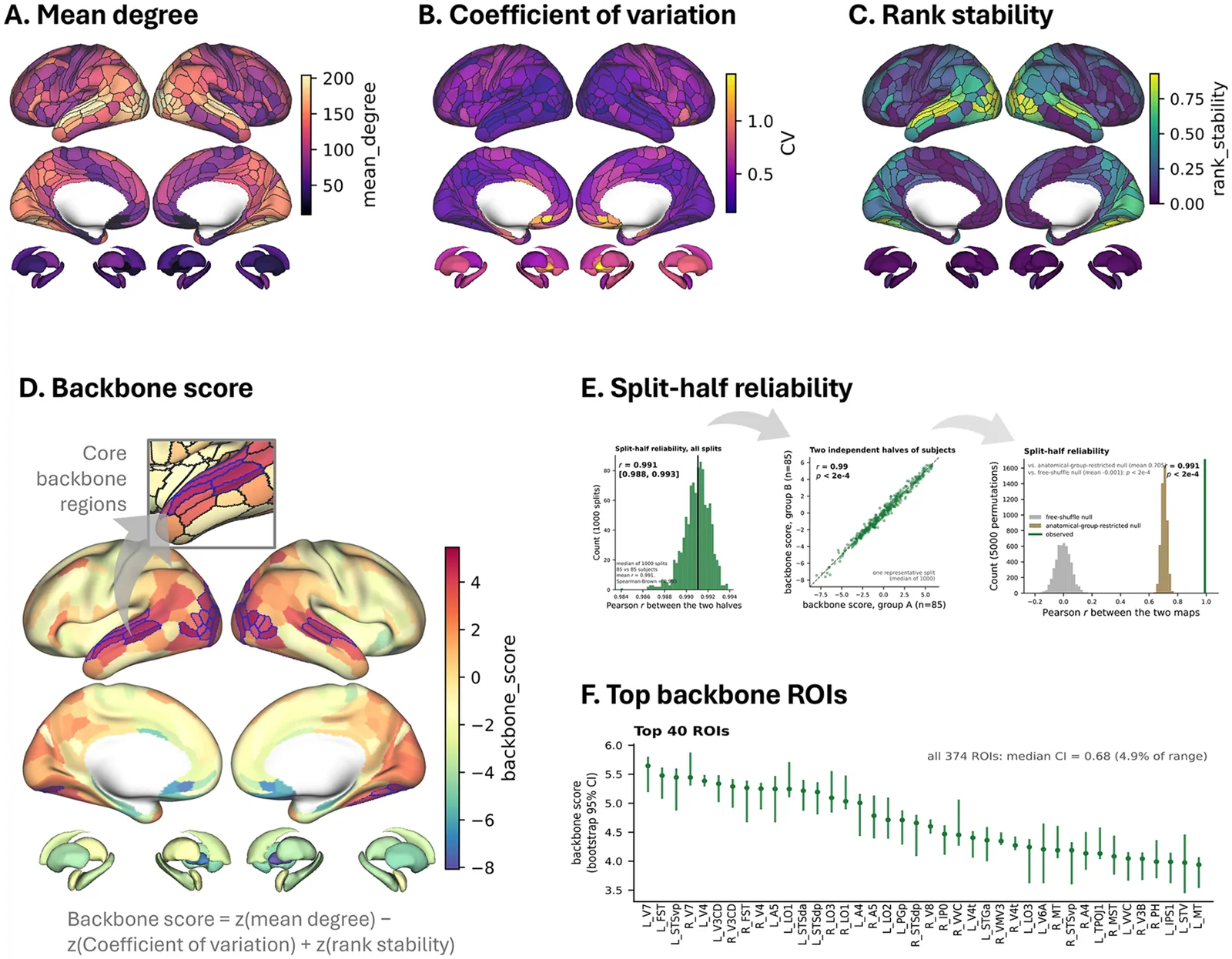
Operational characterization of the conserved degree-centrality backbone. (A) Mean node degree across the 14 movie clips at the primary 30% density threshold. (B) Coefficient of variation (CV) of node degree across clips, indexing cross-clip variability; lower CV indicates greater stability. (C) Rank stability, defined as the fraction of clips in which each ROI fell within the top quartile of node degree. (D) Operational backbone score, computed as Backbone score = z(mean degree) − z(Coefficient of variation) + z(rank stability). Blue outlines indicate high-backbone-score core regions (regions meeting all three core criteria (mean degree top 25%, CV below median, rank stability ≥ 0.75). (E) Whole-pipeline subject split-half reliability of the backbone-score map across 1000 random splits. The representative split-half correlation exceeded the anatomical-group-restricted ROI-label permutation null distribution (r = 0.991,p < 0.001; 5000 permutations). (F) Top backbone-score ROIs with bootstrap confidence intervals from whole-pipeline subject-level resampling.

To characterize the degree-based backbone, we generated ROI-wise maps of mean degree, cross-clip coefficient of variation (CV), and rank stability across the 14 clips. Across all clips, node degree showed a highly consistent spatial distribution, with consistently elevated values in occipital cortex, dorsal and ventral visual streams (DVS and VVS), the temporo-parieto-occipital junction (TPOJ), and bilateral superior and middle temporal gyri (STG/MTG), whereas anterior and medial higher-order association regions showed comparatively lower node degree (Fig. 3A). Importantly, this conserved topography did not imply identical degree values across clips. Rather, regional degree magnitude varied with stimulus context, while the overall posterior-to-anterior organization remained broadly preserved. For example, STG/MTG degree was higher in dialogue-rich clips and lower in clips with little or no speech, such as Movie 3 clips 1 and 4, yet these regions still maintained higher degree than many anterior association regions. CV and rank-stability maps further showed that posterior occipito-temporal regions not only had high degree, but also relatively low cross-clip variability and repeated high-degree ranking across clips (Fig. 3B,C). The variability was greater in orbitomedial frontal and parahippocampal regions, whereas occipital and posterior temporal regions exhibited comparatively lower variability across clips. This finding suggests that different brain regions expressed different levels of sensitivity to clip-specific content within a conserved node degree-based network backbone.

We then summarized these complementary properties using a continuous backbone score (Fig. 3D, and across network-density thresholds see Supplementary Fig. 4). Higher backbone scores reflected regions with high average degree, low cross-clip variability, and high rank stability (see Supplementary Fig. 7). The resulting backbone-score map showed that high-backbone-score regions were concentrated in posterior occipito-temporal cortex, including visual association areas, the MT+ complex, and superior temporal/STS regions. Whole-pipeline split-half analysis showed strong reliability of the backbone-score topography across independent participant subsets. Across 1000 random split-half iterations, map similarity was consistently high (median r = 0.991, central 95% split interval [0.988, 0.993]; Fig. 3E). The median split-half correlation exceeded the anatomical-group-restricted permutation null distribution (r = 0.991,p < 0.001; 5000 permutations; Fig. 3E). This pattern was also stable across network-density thresholds and in exhaustive clip split-half analyses, indicating that the backbone topography was not driven by a single threshold choice (see Supplementary Fig. 6) or by a specific subset of clips (see Supplementary Fig. 5). Bootstrap estimates showed narrow confidence intervals for the top backbone ROIs, supporting the stability of the highest-scoring regions (Fig. 3F). Together, these results indicate a reproducible degree-centrality backbone during movie watching.

### 3.4. Stimulus-dependent modulation of the node degree-based backbone aligns with latent movie feature dimensions

**Fig. 4.**
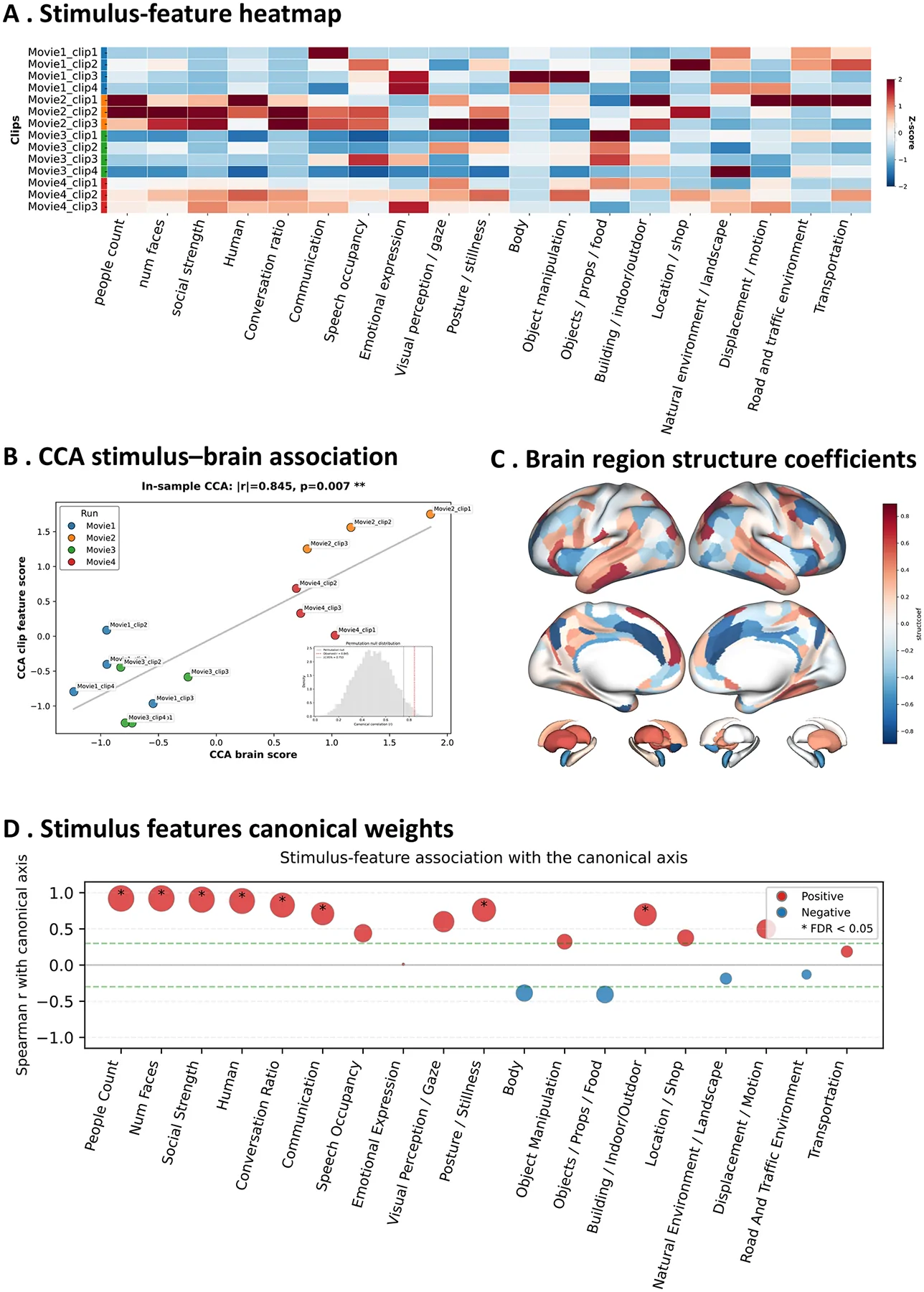
Exploratory association between movie features and degree-map organization. (A) Heatmap of z-scored stimulus features across the 14 movie clips, showing variation in visual, auditory, semantic, and social dimensions. (B) PCA–CCA association between stimulus-feature and brain degree-map representations. Each point represents one clip, colored by movie run. The inset shows the full-pipeline permutation null distribution. (C) Brain structure coefficient map, computed as the correlation between each ROI’s degree values and the brain canonical score across clips. (D) Stimulus-feature associations with the canonical axis, quantified as Spearman correlations between each feature and the stimulus canonical score. Asterisks indicate features surviving FDR correction.

We next examined whether clip-to-clip variability in the organization of node degree maps was associated with systematic differences in stimulus features. To characterize moment-to-moment stimulus structure, we leveraged TR-resolved semantic feature annotations from the Human Connectome Project, which were generated using WordNet-based labeling of visual content. For visualization, we summarized the WordNet-based visual labels using word clouds (Supplementary Fig. 8A). In addition, we extracted speech and dialogue markers from the audio track using pyannote, and quantified the number of people appearing in each segment using YOLO-based detection. Together with eye-gaze annotations from the visual features, these measures were used to derive a time-resolved index of social interaction intensity (Supplementary Fig. 8B). These clip-level features provided a multidimensional description of clip content spanning both non-social visual content (e.g., objects, locations, and motion-related categories) and human-related cues (e.g., faces, speech, and social strength) (Fig. 4B; Supplementary Fig. 9). These features enabled systematic analyses linking naturalistic stimuli to graph-theoretical metrics (specifically node degree) within clip-specific functional connectomes, i.e., stimulus-brain association.

To test this stimulus-brain association, we reduced both the stimulus-feature matrix (14 clips × 19 features) and the brain node-degree matrix (14 clips × 374 ROIs) using principal component analysis (PCA), retaining the first two components on each side (Supplementary Fig. 10) to obtain a low-dimensional representation appropriate for the limited number of clips. Using canonical correlation analysis (CCA), we next assessed multivariate associations between the resulting stimulus and brain scores. At the primary 30% network density threshold, the first canonical mode showed a strong in-sample stimulus–brain association (r = 0.845, full-pipeline permutation p = 0.0078; Fig. 4B). This association remained similar when the full pipeline was repeated across proportional network densities from 10% to 60% (|r| = 0.820–0.861, all p < 0.015; Supplementary Fig. 11). Leave-one-clip-out validation remained positive but did not reach significance under the same full-pipeline permutation framework (5000 permutations at each density threshold; Supplementary Fig. 11). This analysis provided exploratory evidence for a low-dimensional association between stimulus content and clip-level network organization. Movie 3, clip 4 had the largest influence on cross-validated performance (Supplementary Fig. 11).

We characterized the canonical brain pattern using structure coefficients, computed as the correlation between each ROI’s clip-level node degree and the canonical brain score. After orienting the canonical axis so that positive stimulus scores reflected human/social-rich content, positive brain coefficients were most evident in dorsal visual–parietal and attention-related regions, with additional involvement of inferior frontal and temporal cortex. Negative coefficients were more prominent in medial/frontopolar prefrontal, anterior cingulate/medial frontal, frontal opercular/anterior insula, limbic/subcortical, and ventral visual-temporal regions (Fig. 4C). We then examined which stimulus features were significantly associated with the canonical stimulus score using Spearman correlations with FDR correction. Significant positive associations were observed for human- and social-related features, including people count, number of faces, social strength, human-related labels, conversation ratio, and communication. Posture/stillness and building/indoor–outdoor features were also significantly positively associated with the canonical axis (FDR q < 0.05; Fig. 4D and Supplementary Fig. 12). Thus, the first canonical mode distinguished socially rich, human-centered clips from more object- or scenery-dominant clips.

### 3.5. Rich-club systems in large-scale networks

Building on this conserved node degree-based backbone, we estimated which regions act as integrative hubs in movie watching and whether hub organization differs across stimuli. For each movie clip, we computed the node degree–based rich-club architecture from the group-averaged ISFC matrix. Rich-club organization refers to the tendency of high-degree nodes to form densely interconnected cores that facilitate global communication within brain networks (Bullmore and Sporns, 2012). In our study, rich-club regions were defined as nodes whose degree exceeded the k value at which the normalized rich-club coefficient (ϕ_norm(k)) reached its maximum within the statistically significant range (k > max_degree /2; ϕ_norm(k) > 1, p < 0.001, permutation test, Bonferroni corrected; Supplementary Fig. 13).

**Fig. 5.**
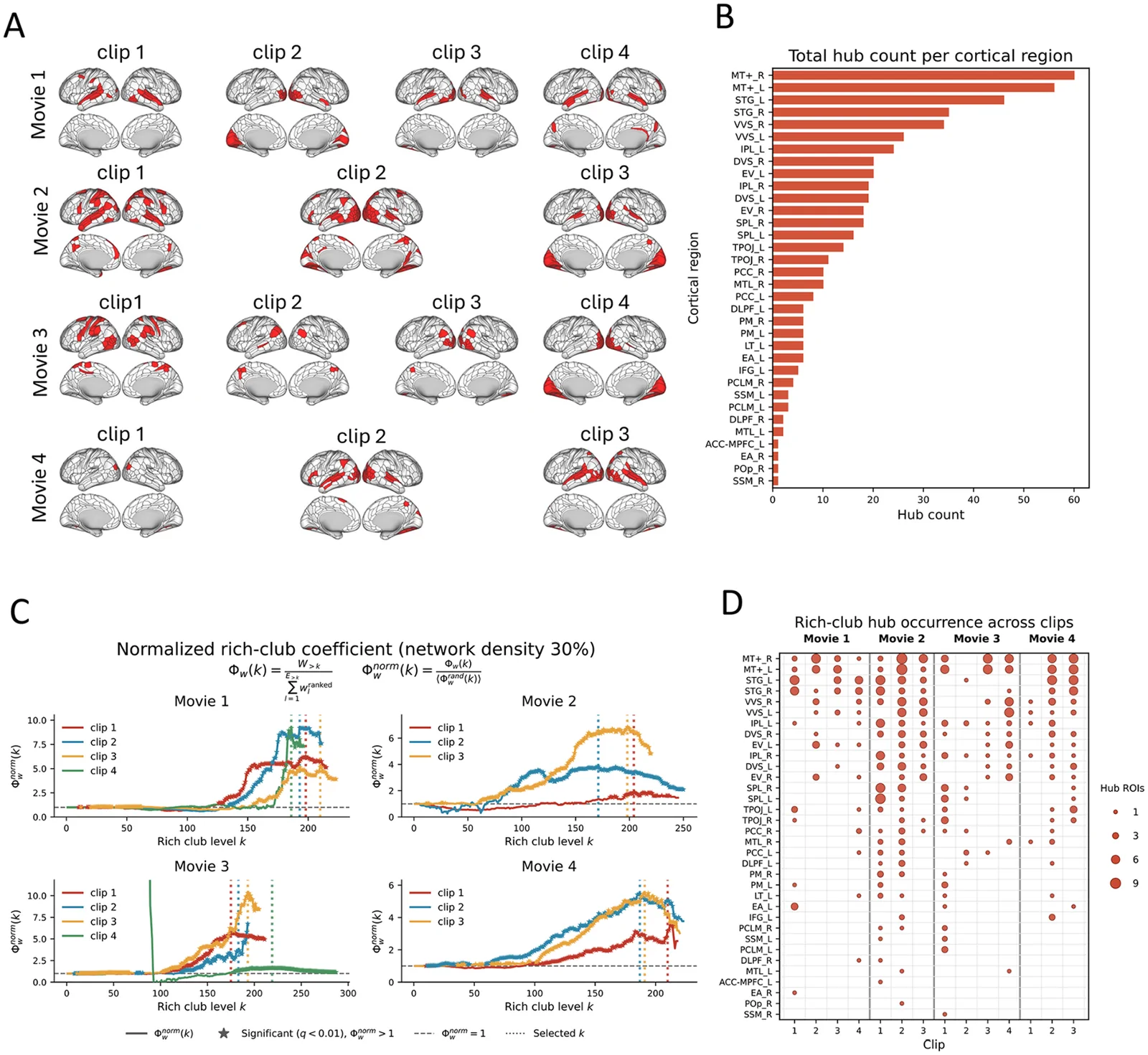
Flexible recruitment of rich-club hubs during movie watching. (A) Rich-club hub regions (marked in red) identified for each movie clip based on node degree-based rich-club analysis of group-averaged inter-subject functional correlation (ISFC) networks (p < 0.01, Bonferroni corrected). (B) Rich-club hubs consistency across cortical anatomical region group. Bar plot shows the cumulative hub count across clips for each cortical system/region, computed as the sum of rich-club subregions identified within each cortical area, highlighting systems that most consistently contributed to rich-club hubs during movie watching (e.g., MT+, STG, and posterior association cortex). (C) Normalized weighted rich-club coefficient, (ϕ_norm(k)) across rich-club levels (k) for each movie clip. (D) Rich-club hub occurrence across clips. Rows indicate cortical regions and columns indicate movie clips, grouped by movie run. Dot size indicates the number of rich-club hub ROIs within each cortical region for each clip.

Across all clips, rich-club hubs showed clip-specific recruitment of subregions within higher-order perceptual and association cortex, most consistently involving posterior temporal, occipital, and parietal regions. As shown in Fig. 5, rich-club regions were consistently identified in bilateral motion-sensitive areas (MT+), STG, inferior parietal lobule (IPL), superior parietal lobule (SPL), DVS and VVS, and TPOJ, with additional involvement of frontal lobe, cingulate cortex, and motor areas in a subset of clips (Fig. 5A–B). Consistent with their proposed integrative role, rich-club regions exhibited high participation coefficients across clips, indicating a predominant connector-hub profile rather than provincial connectivity (Supplementary Fig. 15). Importantly, the specific set of regions identified as rich-club hubs differed across movie clips, with partially overlapping but non-identical combinations of regions engaged in each clip (Fig. 5C). Although the specific regional combinations varied across clips, rich-club organization consistently centered on a predominantly posterior set of perceptual and multimodal integrative regions (e.g., IPL, SPL, TPOJ). We further assessed threshold dependence by repeating rich-club identification across proportional network densities from 10% to 60% (Supplementary Fig. 14). Rich-club membership varied with graph density, with fewer detectable regions at sparse thresholds and more spatially extensive membership at denser thresholds. Thus, we used the 30% density threshold for the primary visualization and provide the full sensitivity maps in the Supplementary Materials.

### 3.6. Rich-club systems show selective connection to large-scale functional networks

**Fig. 6.**
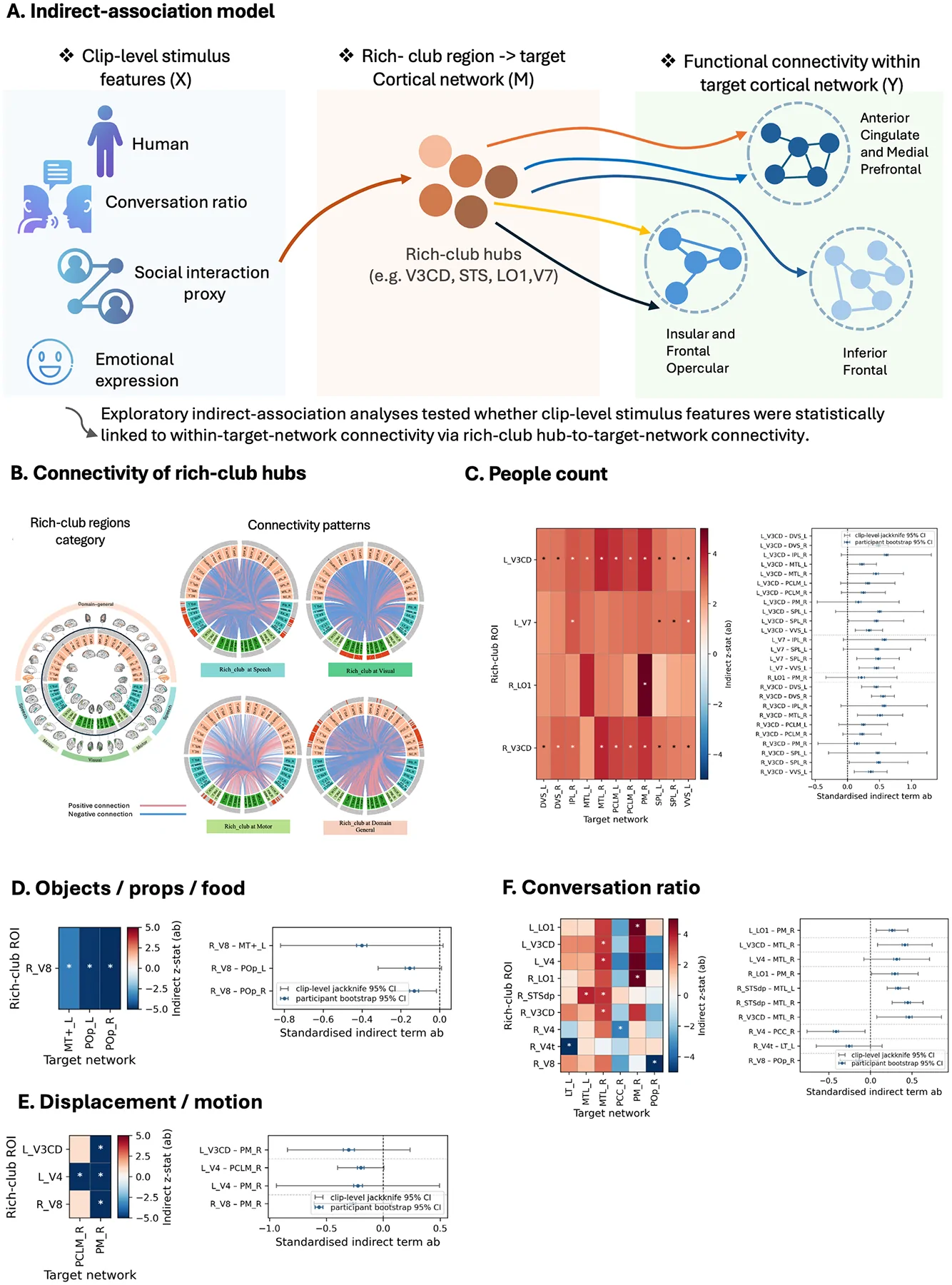
Rich-club pathways differentially mediate stimulus features to cortical networks during movie watching. (A) Conceptual schematic of the mediation framework illustrating the indirect pathway whereby clip-level stimulus features (X) influence within-network functional integration (Y) via rich-club hub–to–target network connectivity (M). Within-network integration was defined as the mean functional connectivity among all subregions within each cortical target system (e.g., anterior cingulate and medial prefrontal cortex, ACC–mPFC; inferior frontal gyrus, IFG). (B) Whole-brain connectivity profiles of rich-club hubs. Rich-club regions were grouped into four functional systems (visual, speech, motor, and domain-general; left). Circular connectivity plots (right) summarize preferential coupling patterns between each rich-club system and distributed cortical targets. (C–F) Representative feature-specific indirect-association results at the primary 30% density threshold. Heatmaps show permutation-based (z)-statistics of the indirect term (ab), with asterisks marking pathways surviving FDR correction (permutation (p < 0.05), FDR-corrected). Forest plots show standardized indirect terms (ab), with participant-bootstrap 95% confidence intervals in blue and delete-one-clip jackknife 95% confidence intervals in gray.

To further elucidate how rich-club hubs interact with the rest of the brain during movie watching, we grouped rich-club regions into four functional systems, i.e., visual, speech, motor, and domain-general systems, based on anatomy and prior functional annotations (Glasser et al., 2016). We then characterized their whole-brain connectivity profiles to assess how different rich-club regions mediated stimulus information to distributed cortical networks (Fig. 6B; Supplementary Fig. 16). Speech-related rich-club hubs showed positive connectivity with language areas and anterior domain-general areas (e.g., the DLPFC and posterior cingulate cortex (PCC)) and negative connectivity with visual cortex. In contrast, visual rich-club hubs showed positive connectivity to visual and posterior domain-general regions (e.g., IPL/SPL and MTL) and negative connectivity to language areas. Motor rich-club hubs showed positive connectivity to motor and visual regions and negative connectivity with language areas. Domain-general rich-club hubs exhibited a functional dissociation: parietal hubs showed preferential positive connectivity with visual areas, whereas PCC subregions preferentially positively correlated with language areas, suggesting system-specific rich-hub organization during naturalistic processing.

### 3.7. Rich-club pathways differentially mediate stimulus features to cortical networks

To explore whether clip-specific stimulus features, rich-club hub-to-target connectivity, and within-target-network functional connectivity showed structured indirect statistical associations, we used a mediation-inspired exploratory path-analysis framework (Fig. 6A; Methods). Rather than testing causal mediation, this analysis was used to characterize feature-dependent indirect-association patterns across rich-club hub × target-network pathways. We focused on indirect-association pathways that survived permutation testing and FDR correction (p < 0.05, FDR-corrected; Methods). For interpretability, Fig. 6 shows the representative stimulus domains, whereas results for the full feature set are provided in Supplementary Fig. 17. Overall, human- and social-communication-related features showed the broadest indirect-association profiles. For example, People count was associated with distributed positive pathways involving visual rich-club hubs, including V3CD, V7, and LO1, and target networks spanning dorsal visual, medial temporal, posterior midline, premotor, superior parietal, and ventral visual systems (Fig. 6C). Conversation ratio showed a similarly distributed but mixed-sign profile, involving occipito-temporal rich-club hubs and lateral temporal, medial temporal, posterior cingulate, premotor, and posterior opercular target networks (Fig. 6F). This pattern was also evident in the full-feature analysis, where human presence, social strength, and speech occupancy showed more distributed pathways than most non-social features (Supplementary Fig. 17). By contrast, object-, motion-, and scene-related features showed more selective indirect-association profiles. Object-related content, indexed by objects/props/food, showed predominantly negative pathways centered on the right V8 rich-club hub and target networks including medial temporal and posterior opercular systems (Fig. 6D). Displacement/motion showed selective pathways involving visual rich-club hubs, including V3CD, V4, and V8, and posterior cingulate/medial or premotor target networks (Fig. 6E). Other features, including body, road and traffic environment, building/indoor/outdoor, and posture-related content, showed comparatively sparse pathways in the full-feature analysis (Supplementary Fig. 17).

### 3.8. Connectome topological feature verification using an in-house dataset

**Fig. 7.**
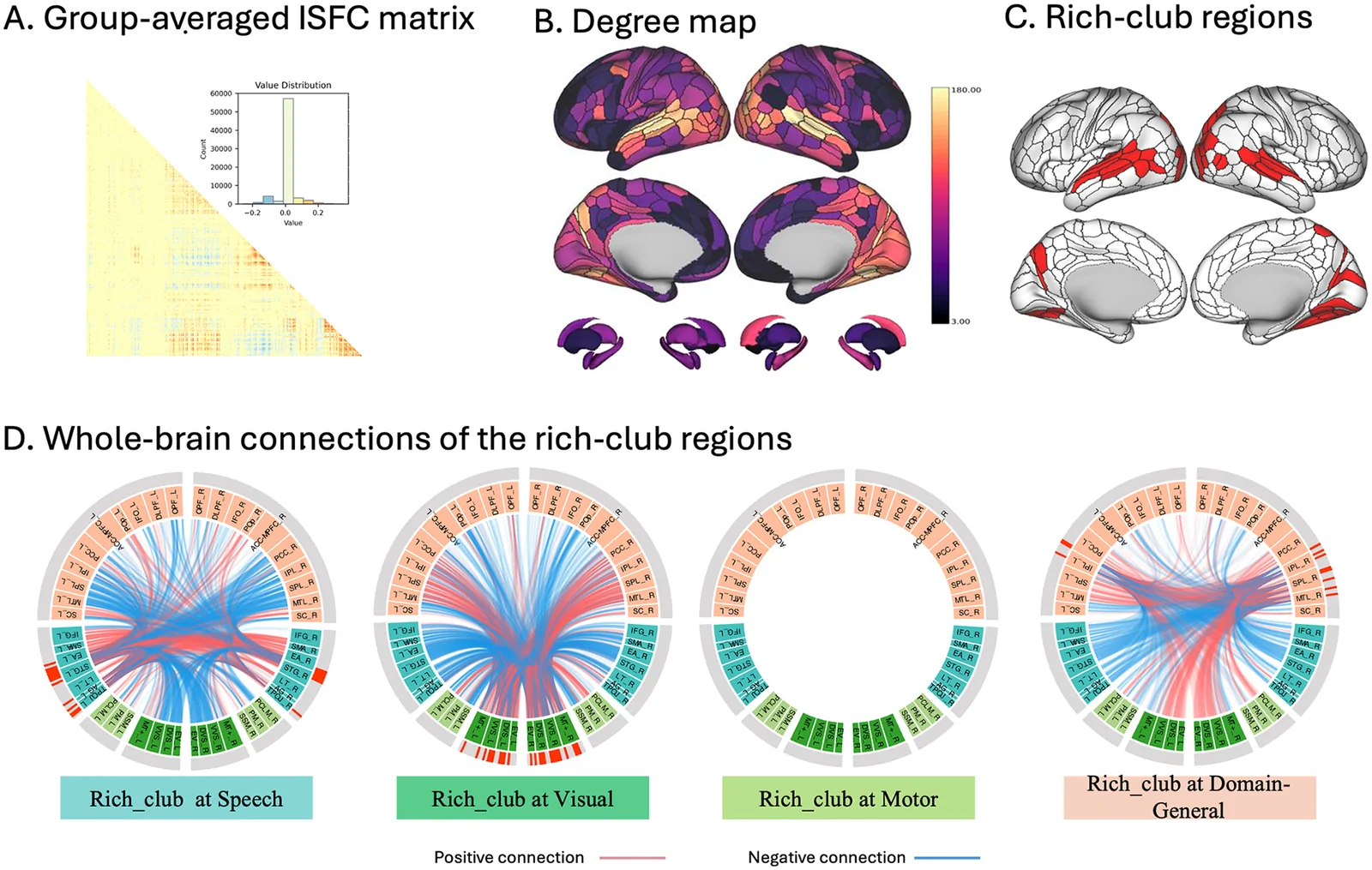
Rich-club organization in a 7-min movie-watching fMRI dataset. (A) The group-averaged ISFC matrix after removing false-positive connections through a t-test (p < 0.01, FDR corrected). (B) Node degree map. (C) Rich-club regions that have the highest normalized rich-club coefficient ⌽norm (p < 0.001, Bonferroni corrected). (D) Whole-brain connections of the rich-club regions.

To validate the connectome topology observed in the HCP dataset, we analyzed an in-house movie-watching fMRI dataset with a stimulus of a 7-minute movie clip (Tie et al., 2015) using the same network analysis pipeline. Higher node degree and clustering were observed in STG, MTG, parietal, and occipital cortices (Fig. 7B). Rich-club organization involved bilateral temporal regions, TPOJ, ventral and dorsal visual streams, SPL, parahippocampus, and PCC (Fig. 7C). Connectivity profiling showed language-related rich-club regions preferentially positively connected with temporal and frontal areas, whereas visual and parietal rich-club regions showed stronger within-network positive connectivity and negative connectivity with language areas (Fig. 7D).

## 4. Discussion

This study reveals a dual-architecture principle underlying large-scale brain connectomic organization during naturalistic movie watching, in which stability and stimulus flexibility coexist. Specifically, movie watching consistently engages a conserved, regional node degree-based network backbone, with the strongest and most stable backbone expression concentrated in posterior temporal, lateral occipital, and adjacent parietal cortices. Rather than reflecting whole-brain remodeling of network topology, clip-to-clip differences primarily reflected selective reweighting of integrative participation by association regions within this conserved backbone, tightly coupled to latent dimensions of stimulus content. In parallel, rich-club analyses identified posterior-dominant integrative hubs whose hub–network connectivity varied with stimulus content, and exploratory mediation analyses suggested that these hubs may link stimulus features to distributed cortical network organization. Together, these findings suggest that naturalistic cognition is supported by a conserved posterior perceptual–association backbone, upon which stimulus-dependent processing is implemented through flexible reweighting of regional integration and rich-club hub organization.

### 4.1. Global network topology

At the global level, global efficiency and modularity followed consistent trends across density thresholds, consistent with the principle of economical small-world organization that balances integration and segregation at a relatively low wiring cost (Bullmore and Sporns, 2012). Nevertheless, substantial differences in these metrics across clips under identical thresholds indicate that stimulus content modulates connectivity weighting within a constrained topology. This aligns with prior work showing partial cross-movie similarity in functional connectivity and activity alongside stimulus-specific patterns (Hasson et al., 2010; Leipold et al., 2024; Tian et al., 2021; Ye et al., 2023).

### 4.2. Conserved degree-based backbone

At the regional level, although individual regions exhibited substantial variability in absolute node degree, the relative spatial distribution of node degree remained highly reproducible across clips. The persistence of this high cross-clip degree-map similarity suggests that naturalistic movie watching engages a conserved network organization, within which diverse movie clips modulate the strength of regional integration. We refer to this conserved organization as a degree-based backbone: a reproducible degree-centrality topography in which a set of regions consistently occupies central positions across diverse movie contexts. The backbone score further summarized this organization as a posterior-to-anterior degree-centrality pattern: regions with the highest scores were concentrated in posterior parietal, dorsal and ventral visual streams, MT+ complex, and temporal regions, whereas anterior and medial higher-order regions showed lower and more context-dependent involvement. Importantly, whole-pipeline split-half, bootstrap, permutation, cross-density, and clip-partition analyses supported the reliability of this posteriorly centered backbone, indicating that the observed topology was not driven by a single participant sample, threshold choice, or subset of movie clips.

The highest backbone-score regions were concentrated in posterior perceptual and adjacent association cortices, including lateral occipital, posterior parietal, and lateral temporal regions. These regions also showed high rank stability, indicating that they repeatedly occupied high positions in the node-degree hierarchy across movie contexts. These perceptual and adjacent association regions play central roles in audiovisual decoding, semantic processing, and multisensory integration during naturalistic stimulation (Beauchamp, 2005; Beauchamp et al., 2004; Huth et al., 2016). Their stability likely reflects strong anatomical constraints and shared stimulus-driven synchronization, consistent with extensive neurocinematics evidence demonstrating robust inter-subject correlation in auditory and visual cortices during movie watching (Golland et al., 2007; Hasson et al., 2004; Li et al., 2025). In contrast, medial/orbitofrontal PFC, ACC, para-hippocampal gyrus, and subcortical regions implicated in higher-order cognition processing (Lieberman, 2007) showed systematically lower backbone-score and higher cross-clip variability, and lower rank stability, suggesting more context-dependent engagement. These findings provide connectome-level support for prior work showing that higher-order association systems flexibly adjust their engagement depending on naturalistic stimulus demands (Demirtaş et al., 2019; Hasson et al., 2008; Li et al., 2025; Simony et al., 2016; Ye et al., 2023). Together, these findings suggest that the degree-based backbone provides the stable component of a dual-architecture framework, on which stimulus-dependent reweighting and flexible hub reconfiguration can occur.

### 4.3. Stimulus-dependent reweighting of the conserved degree-based backbone

Given the clip-dependent variability in node degree superimposed on a conserved backbone, we tested how this reweighting systematically maps onto stimulus features. CCA between PCA-derived brain node degree score maps and PCA-reduced stimulus features provided exploratory evidence for a low-dimensional association between clip-level node-degree organization and stimulus-feature profiles, suggesting that selective reweighting of the conserved backbone was not arbitrary but tracked meaningful dimensions of naturalistic content. This finding extends models of large-scale network organization that emphasize stable architectural constraints alongside context-dependent reconfiguration (Deco et al., 2011) into an ecologically valid naturalistic domain (Sonkusare et al., 2019). We characterized the canonical brain pattern at a broader systems level. Positive brain coefficients were most evident in dorsal visual–parietal and attention-related regions, with additional involvement of inferior frontal and temporal cortex. These systems have been implicated in visuospatial attention and perceptual selection, language and semantic integration, and action- or social-perceptual processing (Adolphs, 2001; Corbetta and Shulman, 2002; Deuse et al., 2016; Hagoort, 2019). Negative coefficients involved medial frontopolar prefrontal, anterior cingulate/medial frontal, frontal opercular/anterior insula, limbic/amygdala, and ventral visual-temporal regions. These regions have been associated with internal-state integration, affective and motivational salience, interoceptive awareness, valuation, and self-related processing (Bechara et al., 2000; Di Plinio et al., 2020; Haber and Knutson, 2010). Together with the significant associations between the canonical stimulus axis and human/social-related features, these findings suggest that socially rich and human-centered clips were associated with systematic variation in node-degree organization across distributed perceptual, attentional, language-related, social-perceptual, and affective/interoceptive systems.

Together, these results suggest that clip-level stimulus features were associated with systematic variation in regional node-degree organization along a canonical brain axis that was broadly consistent with the conserved degree-based backbone. Thus, stimulus-dependent reweighting appears to occur within a stable degree-centrality hierarchy rather than through arbitrary reorganization of the functional connectome. Because the effective sample size was limited to 14 clips and leave-one-clip-out performance was positive but not significant, this result should be interpreted as an exploratory in-sample association between stimulus features and node-degree organization, rather than as evidence for a validated out-of-sample predictive model.

### 4.4. Flexible rich-club hub organization

Extending the dual-architecture framework, rich-club analysis identified a posterior-dominant rich-club organization that provides a mechanistic substrate for stimulus-dependent reweighting of large-scale brain networks during movie watching. Rich-club regions identified in this study include higher-order visual cortex (VVS / DVS and MT+), temporo-parietal association cortex (STG and TPOJ), and dorsal parietal regions (IPL/SPL), with additional contributions from midline integrative regions such as the precuneus and PCC. These posterior hubs have consistently shown high synchronization of activity across subjects during movie watching (Chen et al., 2017; Hasson et al., 2004; Nummenmaa et al., 2012), with varying node degrees of spatial extent across different movie types (Hasson et al., 2008; Leipold et al., 2024).

Importantly, rich-club organization was not fixed across clips. Instead, hub composition and connectivity strength of rich-club hubs varied with stimulus demands. Clips rich in speech or dialogue preferentially recruited STG and TPOJ as rich-club hubs, highlighting their role as integrative nodes for spoken language processing and social-cue integration during naturalistic communication (Redcay and Warnell, 2018). Clips featuring complex spatial transitions additionally recruited parietal and motor regions that were involved in spatial updating and intention inference (Hamilton and Grafton, 2006; Rolls, 2023), and in some cases recruited higher-order areas such as left IFG and DLPFC. Our results show that rich-club composition varies with stimulus demands, indicating that hub regions flexibly adjust their spatial distribution and connectivity strength on top of a conserved degree-based backbone. This extends prior work on stimulus-specific activation patterns across different movies (Hasson et al., 2008).

Mapping rich-club hubs onto language, visual, motor, and domain-general systems revealed a structured pattern of integration and segregation. Speech-related hubs showed preferential coupling with language and domain-general systems, whereas visual-related hubs were more strongly embedded within visual–parietal systems and showed reduced between-network connectivity. This organization is consistent with canonical principles of functional segregation and integration in the human brain (Bertolero et al., 2015; Fox et al., 2005; Wang et al., 2021), suggesting that rich-club hubs may support content-specific routing of speech and visual information within the conserved backbone architecture.

### 4.5. Exploratory indirect associations involving rich-club hub connectivity

The mediation-inspired exploratory path analysis revealed structured indirect associations among clip-level stimulus features, rich-club hub-to-target connectivity, and within-target-network functional connectivity. Specifically, stimulus features were associated with network integration through hub-to-target pathways from temporal, parietal, and higher-order visual regions. These findings are consistent with prior evidence showing that posterior temporal and parietal regions support multisensory audiovisual integration (Beauchamp, 2005) and semantic and narrative processing during movie watching (Hasson et al., 2008). The observation that each stimulus feature showed indirect-association through multiple hub–to–target network pathways toward different cortical networks supports the view that naturalistic processing relies on coordinated interactions across distributed brain systems (Zhang et al., 2021).

Human-, social-, and conversation-related features showed the broadest indirect-association profiles, involving distributed visual, temporal, parietal, posterior cingulate/medial, premotor, and medial temporal systems. These networks are implicated in language comprehension, social inference, executive control, memory integration, and event representation (Baldassano et al., 2017; Friederici, 2011; Hasson et al., 2008; Miller and Cohen, 2001; Spreng and Andrews-Hanna, 2015). These findings may suggest that, under naturalistic conditions, the integration of social and human-related information involves coordinated processing across multiple higher-order cortical systems, potentially supported by rich-club hub-to-target connectivity during complex social interpretation. By contrast, object- and motion-related features showed more focal and sign-specific profiles, broadly consistent with prior work showing object recognition relies on specialized ventral visual and semantic pathways (Grill-Spector and Weiner, 2014; Haxby et al., 2001). Given the correlational design, clip-level stimulus predictors, and limited number of clips, these findings should be interpreted as exploratory, hypothesis-generating indirect associations rather than evidence for causal or mechanistic mediation.

### 4.6. Topology-level validation in an independent movie-watching dataset

To test the generalizability of the connectome backbone, we repeated the degree and rich-club analyses in our in-house movie-watching fMRI dataset with a single continuous 7-min movie clip as the stimulus. This longer stimulus minimizes potential carryover effects across clips and provides a complementary test of topological stability under sustained naturalistic viewing. Based on the in-house dataset, we observed a convergent backbone closely matching that in the HCP data. This provides partial, topology-level validation: it supports the robustness of temporo-occipital and parietal association regions as a stable integrative backbone. External validation of the stimulus-feature CCA and mediation analyses will require further study.

### 4.7. Exploratory effects of movie content on backbone engagement

On an exploratory basis, and given the small number of clips per film category, we further considered which types of movie content most reliably engage the connectome backbone across individuals. We observed that Hollywood films did not uniformly outperform other stimuli. Specifically, characteristics and rich-club organization in independent films were comparable, and one Hollywood clip (Movie 4, clip 1) exhibited weaker organization than an independent film clip. Nevertheless, several Hollywood clips (e.g., Movies 2 and 3, clips 2–3) showed high pairwise similarity, likely reflecting shared narrative structure, interpersonal focus, and a clear central character (Bordwell, 2006; Hasson et al., 2008). Consistent with prior work showing that abstract, nonverbal stimuli resemble resting-state connectivity responses (Kröll et al., 2023), clips lacking dialogue or narrative structure elicited weaker topology and predominantly visual organization. In contrast, socially and narratively rich clips, across both Hollywood and independent films, produced the strongest and most reliable rich-club recruitment, particularly within temporal, parietal, and higher-level visual systems. Together, these findings tentatively suggest that stimuli rich in socially and behaviorally meaningful content may most reliably elicit stable and reproducible backbone configurations across individuals, consistent with a content-dependent constraint on the stability of large-scale network topology during naturalistic movie watching.

## 5. Limitations

Several limitations should be noted in this study. First, the number of clips was modest relative to the dimensionality of semantic features, which may bias multivariate alignment toward the dominant scene-–object axis. Larger stimulus sets will be needed to more fully dissociate semantic dimensions, including social and affective factors. Second, although we replicated the degree-based backbone and rich-club organization in an in-house dataset with a longer and continuous stimulus, the convergence of these patterns despite differences in scanner field strength, TR, voxel size, preprocessing, stimulus duration and content, and sample size is encouraging, suggesting that the broad topology is not specific to a single acquisition protocol or stimulus set. However, these differences also introduce methodological heterogeneity. This in-house dataset did not include matched semantic feature annotations, preventing an external validation of the feature-alignment and mediation analyses. Future work combining longer continuous stimuli with time-resolved semantic labeling will be essential for testing whether the same stimulus dimensions drive backbone reweighting across datasets. Third, mediation analyses quantify statistical indirect pathways but do not establish causal directionality, motivating future work using effective connectivity models or perturbational approaches. Fourth, because rich-club hubs were defined from node degree-based topology under thresholded connectivity, future analyses should evaluate parameter sensitivity and extend to weighted or multilayer formulations. Movie clips were presented in a fixed order with only 20-s rest periods between clips. Thus, clip-level differences may partly reflect order, fatigue, adaptation, or carryover effects in addition to stimulus-content differences. Following (Finn and Bandettini, 2021), we assigned the first 5 TRs after each clip to the preceding clip to account for the delayed BOLD response and excluded the remaining 15 TRs of the rest period from clip-level ISFC estimation. However, we did not systematically test alternative lag lengths or rest-exclusion windows. Therefore, residual hemodynamic carryover cannot be fully excluded, and clip-level findings should be interpreted cautiously. Despite these limitations, the convergence across clips and with an independent dataset supports the reproducibility of a broad posterior degree-centrality topology and associated rich-club organization. In contrast, the stimulus-feature alignment and mediation analyses should be interpreted as exploratory evidence for possible content-related modulation of this topology. On the other hand, global signal regression and spatial smoothing can influence functional connectivity estimates and graph-theoretical measures. Thus, the reported topology should be interpreted in the context of the preprocessing choices used here, and future studies should further evaluate optimal preprocessing strategies for movie-watching fMRI analyses.

## 6. Conclusion

The findings of this study demonstrate that naturalistic cognition is supported by a dual-architecture principle of large-scale brain networks: a conserved node degree-based backbone ensures reliable audiovisual integration, while higher-order association regions flexibly reweight their participation according to stimulus content. Variability within this backbone is systematically aligned with socially and behaviorally salient features, and is implemented through rich-club–mediated interactions across functional systems. Together, these findings provide a mechanistic account of how stability and flexibility are balanced in the human connectome during movie watching.

## Supplementary Material

1

Supplementary material associated with this article can be found, in the online version, at doi:10.1016/j.neuroimage.2026.122171.

## Data Availability

The data used in this study are publicly available from the Human Connectome Project (HCP) database. Access to the HCP dataset requires registration and adherence to the data use terms (https://www.humanconnectome.org/). The in-house dataset and custom code used for data processing and analysis are available from the corresponding author upon reasonable request.
