# Supplementary material for: A conserved node degree-based backbone and flexible hub organization of brain connectome during naturalistic movie watching
Source: Neuroimage. Author manuscript; Available in PMC 2026 Sep 21. (PMC13591665; doi:10.1016/j.neuroimage.2026.122171)
Supplement: Supplementary file 1 [file NIHMS2210256-supplement-1.docx]

**Supplemental information**

**A conserved node degree-based backbone and flexible hub organization of brain connectome underlying naturalistic movie watching**

Xuehu Wei^1,2^, Laura Rigolo^1^, Colin P. Galvin^1^, Einat Liebenthal^2^, Yanmei Tie^1*^

1.	Department of Neurosurgery, Brigham and Women’s Hospital, Harvard Medical School, Boston, Massachusetts, USA

2.	McLean Imaging Center, McLean Hospital, Harvard Medical School, Belmont, Massachusetts, USA

***Corresponding author**

**Email:** [**ytie@bwh.harvard.edu**](mailto:ytie@bwh.harvard.edu)

**Supplemental method**

**MRI data**

Movie-watching fMRI data were acquired on a 7.0 Tesla Siemens Magnetom scanner Functional blood oxygen level-dependent (BOLD) images were acquired in a single-shot gradient-echo echo-planar imaging (EPI) with a Nova32 32-channel head coil: TR = 1000 ms, TE = 22.2 ms, flip angle = 45°, field of view (FOV) = 208 × 208 mm, in-plane resolution = 130 × 130, voxel size = 1.6 mm isotropic, 85 axial slices, multiband acceleration factor = 5, in-plane acceleration (iPAT) = 2, partial Fourier = 7/8, echo spacing = 0.64 ms, and bandwidth = 1924 Hz/Px. fMRI data were acquired with alternating phase-encoding directions: Movie 1 and Movie 3 in the anterior–posterior (AP) direction, and Movie 2 and Movie 4 in the posterior–anterior (PA) direction. The total durations of the four movie runs were 921 sec, 918 sec, 915 sec, and 901 sec, respectively.

Movie-watching fMRI data were acquired on a 3.0 Tesla GE Signa system (General Electric, Milwaukee, WI, USA). fMRI images were acquired in a single-shot gradient-echo EPI and a standard quadrature head coil: TR= 2000 ms, TE = 40 ms, flip angle = 90°, slice gap = 0 mm, FOV = 256 × 256 mm, in-plane resolution = 128 × 128, voxel size = 2 × 2 × 4 mm^3^, 27 axial slices, ascending interleaved sequence. Structural images were acquired using a T1-weighted 3D spoiled gradient recalled (SPGR) spatial sensitivity encoding technique (ASSET, i.e., parallel imaging) and an 8-channel head coil: TR= 7.8 ms, TE = 3.0 ms, flip angle = 20°, in-plane resolution = 512 × 512, voxel size = 0.5 × 0.5 × 1 mm^3^, 176 axial slices.

**Graph-theoretical metrics and rich-club analysis**

*Network construction*

For each movie clip, the group-averaged ISFC matrix $M\in R^{N\times N}$was thresholded at a fixed density. Then graph metrics of a given network matrix M were computed as follows.

*Degree and clustering coefficient of a node*

The degree of a node represents the number of connections (edges) it has to other nodes in the network. The degree $d_{i}$ of a node i is defined as:

$$d_{i}=\sum_{i\neq j} b_{ij}$$

Where**:**

$b_{ij}$: denotes the binary adjacency matrix, where $b_{ij}=1$where if a functional connection exists between nodes $i$and $j$after thresholding, and $b_{ij}=0$ , otherwise.

*Global efficiency*

Global efficiency of the brain network refers to a measure of how effectively information or resources are exchanged across the entire brain. It is defined based on the shortest path lengths between nodes:

$$E_{global}=\frac{1}{N(N-1)}\sum_{i\neq j} \frac{1}{d_{ij}}$$

Where:

$d_{ij}$: Shortest path length (geodesic distance) between regions 𝑖 and 𝑗,

N: the total number of possible node pairs in the network.

*Modularity*

Modularity is a measure of the extent to which a network can be divided into distinct, densely interconnected groups of nodes (i.e., modules or communities) with sparse connections between groups. In this study, we examined the modular organization of brain functional networks using the Louvain algorithm (Rubinov and Sporns, 2010; van den Heuvel and Sporns, 2011).

*Rich-club organization*

We examined the rich-club organization of the brain functional networks by computing the rich-club coefficients ϕ(k) across a range of degrees k (Rubin et al., 2017). The weighted rich-club parameter ϕ^w^(k) was computed as the ratio between the total weight of connections among nodes with degrees greater than k (W_>k_) and the sum of the weights of the strongest E_>k_ connections in the entire network: ϕ^w^(k) is defined as:

$$\phi^{w}(k)=\frac{W_{>k}}{\sum_{i=1}^{E>K} \omega_{i}^{ranked}}$$

where:

E_>k_  is the number of connections among nodes with degrees greater than k, and the weights are derived from the ranked connections in the network.

$W_{>k}$is the total weight of connections among nodes with degrees greater than k,

$\sum_{i=1}^{E>K} \omega_{i}^{ranked}$ is the sum of the weights of the to E_>k_ connections in the network, ranked by their weights.

To assess the statistical significance of the rich-club organization, a permutation testing was employed using a population of 1,000 randomized networks, preserving the degree distribution and sequence of the original network. For each degree k, the weighted rich-club coefficient ϕwrandom(k) was computed for each of the randomized networks. The average rich-club coefficient ϕwrandom(k) was then calculated as the mean value across m random networks. Finally, the normalized rich-club coefficient ϕwnorm(k) was computed as:

$$\phi^{wnorm}(k)= \frac{\phi^{w}(k)}{\phi^{wrandom} (k)}$$

A significant rich-club organization (i.e., ϕ^norm^(k)> 1) indicates that high-degree nodes are more interconnected than chance. Statistical significance was evaluated using Bonferroni-adjusted α-levels of 0.01 per test (**see** ***Supplementary Fig. 13***).

*Rich-club centrality*

We assessed the centrality of the rich-club to evaluate its role in the global network structure. Specifically, rich-club regions were classified as “provincial hubs” if they exhibited a high within-module degree, and as “connector hubs” if they showed a participation coefficient (PC) that is higher than 0.5 (Bullmore and Sporns, 2012). The within-module degree identifies regions that are highly connected within their own functional community, whereas the PC quantifies the diversity of inter-module connections, with values above 0.5 indicating substantial cross-community integration. These measures highlight the extent to which the rich-club region serves as a backbone for both local specialization and efficient global communication across the entire brain.

**Stability and reliability analysis of the degree-centrality backbone**

To assess the stability and reliability of the degree-centrality backbone, we performed whole-pipeline resampling and permutation analyses in participants with usable data across all 14 clips. In each resampling iteration, the full ISFC-to-degree pipeline was recomputed rather than resampling precomputed degree or backbone-score values. For each clip, subject-level raw ISFC (z)-matrices were entered into an edgewise one-sample (t)-test against zero. (P) values were Bonferroni-corrected across all unique edges, and edges with corrected (p > 0.001) were set to zero in the group-mean ISFC matrix. The absolute significant mean ISFC matrix was then proportionally thresholded, binarized, and converted into an unweighted node-degree map. The primary analysis used the 30% density threshold, with sensitivity analyses repeated across 10%–60% densities.

For each ROI, backbone metrics were computed across the 14 clips. A continuous backbone score was computed as (z(mean degree) - z(coefficient of variation) + z(rank_stability)), with each metric standardized across ROIs before combination. ROIs were classified as core top backbone regions if they met three prespecified criteria: top-quartile mean degree, below-median CV, and rank stability (≧ 0.75).

For the bootstrap analysis, participants were sampled with replacement for 1,000 iterations, and the full pipeline was recomputed, and backbone-score maps were compared in each iteration. Bootstrap distributions were used to estimate confidence intervals for ROI-wise backbone scores and selection frequencies for top core backbone-region classification. For split-half reliability, participants were randomly divided into two non-overlapping halves for 1,000 iterations. The full pipeline was recomputed independently within each half, and the resulting backbone-score maps were compared using map-wise correlations across ROIs.

Stimulus-level generalization was assessed using clip-partition and leave-one-movie-out analyses. We first performed an exhaustive 7-versus-7 clip split-half analysis, in which backbone-score maps were computed separately for each half of the clips and correlated across ROIs. We then performed leave-one-movie-out analyses, in which the backbone-score map derived from clips belonging to one movie run was correlated with the map derived from the remaining clips. Because each held-out movie run contained only three or four clips, leave-one-movie-out correlations were compared with exhaustive same-size random clip partitions that ignored movie-run identity.

Permutation-based null models were used to test whether the observed backbone organization exceeded expectations from spatial label shuffling. Map-to-map significance for split-half and leave-one-movie-out backbone-score correlations was also assessed using 5,000 ROI-label permutations, and anatomical-group-restricted results were reported.

**Stimulus feature extraction and clip-level feature representation**

TR-resolved semantic annotations were obtained from the HCP 7T movie-watching dataset. These annotations label the presence of WordNet-based stimulus categories at each time point for every movie clip. For visualization, WordNet labels were summarized using word clouds and feature time courses. The final visual-semantic feature set included Human, Body, Face, Building/indoor/outdoor, Road and traffic environment, Transportation, Natural environment/landscape, Objects/props/food, Location/shop, Displacement/motion, Posture/stillness, Visual perception/gaze, Communication action, Emotional expression, and Object manipulation.

In addition,Speech presence was detected using pyannote (Plaquet and Bredin, 2023), and face-related features were extracted using Pliers (McNamara et al., 2017) At the TR level, we binarized speech and conversation as 0/1 indicators reflecting whether speech or dialogue was present at each TR. At the clip level, we summarized these measures as speech occupancy and conversation ratio, defined as the proportion of TRs labeled as speech or conversation within the clip, respectively. To quantify human presence, we detected people in each frame using YOLO (Martynenko et al., 2025; Redmon and Farhadi, 2018) and computed the number of individuals per TR. To reduce the influence of background bystanders (e.g., distant pedestrians) on social estimates, we restricted social computations to close-range people, defined using bounding-box size and position thresholds: a detection was classified as close-range if its bounding-box area exceeded 0.08 of the frame (close_area_min = 0.08) and its bottom coordinate was below 70% of the frame height (close_bottom_min = 0.7). Conversely, detections with bounding-box area < 0.03 (far_area_max = 0.03) and bottom coordinate < 60% of frame height (far_bottom_max = 0.5) were treated as far-range and excluded from social strength calculations. We further defined a measure of social strength as a composite index of social intensity for each (Martynenko et al., 2025). Specifically, we defined an interaction cue as a binary variable set to 1 when interaction-related WordNet labels (e.g., talk.v.01, conversation.n.01, gaze.v.01, look.v.01, argue.v.01, kiss.v.02, hug.v.01) were present at a given TR, and 0 otherwise. Social strength was then computed as:

social strength = (number of close-range people) × (interaction cue) × (communication).

**Mediation anylysis**

We performed subject-level mediation analyses to test whether rich-club hub connectivity mediates the relationship between movie semantic features and large-scale network integration. Analyses were based on clip-wise functional connectivity from 176 participants across 14 movie clips and were restricted to regions identified as rich-club members in at least 6 clips. Mediation analyses were conducted at the subject level to preserve inter-individual variability in functional connectivity and to avoid inflating statistical evidence through clip-level averaging.

For each subject $s$, clip $c$, rich-club hub $r$, and target sub-netwoek $l$, the mediator and outcome variables were defined as:

$$M_{s,c}^{(r,l)}=\frac{1}{\mid l\mid}\sum_{i\in l} \mid W_{s,c}(r,i)\mid$$

$$Y_{s,c}^{(l)}=\frac{2}{\mid l\mid(\mid l\mid-1)}\sum_{i<j,\text{ }i,j\in l} \mid W_{s,c}(i,j)\mid$$

where $W_{s,c}$denotes the subject-level functional connectivity matrix (top 30% density, Fisher *z*-transformed, tanh-normalized). Analyses focused on absolute edge weights; signed variants yielded comparable results.

For each subject, mediation was estimated across clips using linear models:

$$M_{s,c}=a_{s}X_{c}+\varepsilon_{s,c},Y_{s,c}=b_{s}M_{s,c}+c_{s}^{'}X_{c}+\eta_{s,c},$$

where $X_{c}$ denotes the clip-wise semantic feature value. The subject-level indirect effect was defined as $ab_{s}=a_{s}b_{s}$.

Group-level indirect effects were summarized by bootstrapping across subjects (1,000 iterations; mean and 95% confidence intervals). Statistical significance was assessed using clip-level permutation testing (5,000 permutations of $X_{c}$), yielding permutation-based *p* values and *z* statistics. False discovery rate correction was applied across all rich-club hub and target sub-network pairs.

**Supplementary**

**Supplementary figures**

***Supplementary Fig. 1. Threshold sensitivity of global efficiency and modularity.*** Global efficiency and modularity (Q) were computed for each clip-wise ISFC network across proportional density thresholds from 10% to 60%. Panels show results separately for the four movie runs, with each line representing one clip. The primary 30% density threshold used in the main analyses. Global efficiency generally increased with network density, whereas modularity decreased at higher densities, illustrating the dependence of global graph metrics on threshold choice.

***Supplementary Fig. 2.* Cross-clip similarity of the whole-brain degree pattern exceeds chance at every density.** Cross-clip similarity of node-degree maps was evaluated across proportional network density thresholds from 10% to 60%. Each panel shows the bootstrap distribution from 5,000 whole-pipeline subject-level resamples and the corresponding unconstrained and anatomical-group-restricted ROI-label permutation null distributions. Across all densities, observed similarity was high, bootstrap confidence intervals were narrow, and observed values exceeded both null distributions (all permutation p < 0.001). Similarity decreased only gradually with increasing density, from R = 0.543 at 10% to R = 0.475 at 60%.

***Supplementary Fig. 3.*** **Cross-clip node-degree similarity across network density thresholds.** Pairwise similarity matrices of clip-wise node-degree maps across the 14 movie clips are shown for proportional network density thresholds from 10% to 60%. Each matrix entry represents the Pearson correlation between the node-degree maps of two clips. Color intensity indicates the magnitude of cross-clip degree-map similarity. Mean cross-clip similarity remained moderate across thresholds, with mean (R = 0.543), 0.526, 0.514, 0.502, 0.492, and 0.475 at 10%, 20%, 30%, 40%, 50%, and 60% density, respectively. The 30% density threshold was used as the primary threshold in the main analyses. Asterisks indicate pairwise correlations that exceeded the anatomical-group-restricted ROI-label permutation null after FDR correction. These results show that the conserved node-degree topology was robust across density thresholds.

***Supplementary Fig4.* Threshold sensitivity of backbone metrics and composite backbone score.** Brain maps show mean node degree, coefficient of variation (CV), rank stability, and the composite backbone score across proportional network density thresholds from 10% to 60%. The backbone score was computed as z(mean degree) − z(CV) + z(rank stability), thereby identifying regions with high and stable degree across clips. Across thresholds, posterior occipito-temporal and parietal cortices consistently showed high mean degree, low CV, high rank stability, and high backbone scores.

***Supplementary Fig. 5.* Clip-level generalization and movie-run grouping of the degree-centrality backbone**. (A) Exhaustive 7-versus-7 clip split-half analysis at the primary 30% network density threshold. Each point represents one ROI, comparing backbone scores computed from two non-overlapping sets of seven clips. The displayed split corresponds to the median correlation across all 1,716 unique clip partitions. (B) ROI-label permutation test for the representative clip split-half correlation. The observed map correlation exceeded both the unconstrained free-shuffle null and the anatomical-group-restricted null distribution (r = 0.848, p < 0.001 5,000 permutations). (C) Movie-run grouping analysis comparing backbone scores derived from MOVIE1+MOVIE3 clips versus MOVIE2+MOVIE4 clips at the 30% density threshold. (D) The observed MOVIE1+MOVIE3 versus MOVIE2+MOVIE4 correlation was compared against all size-matched 8-versus-6 clip partitions, showing a lower-than-expected correlation relative to random clip groupings (r = 0.686, exact p = 0.0010). (E) Exhaustive clip split-half correlations across network density thresholds from 10% to 60%. Black bars indicate the median correlation across all 1,716 partitions at each density. (F) Comparison between the MOVIE1+MOVIE3 versus MOVIE2+MOVIE4 grouping and the distribution of exhaustive clip split-half correlations across densities. The movie-run grouping correlation fell below the 95% interval of random size-matched clip partitions at all tested densities, suggesting that backbone-score topography was generally stable across clips but showed reduced similarity between these movie-run subsets.

***Supplementary Fig. 6.* Cross-density robustness of the degree-centrality backbone**. (A) Whole-pipeline subject split-half reliability of the backbone-score map across network density thresholds from 10% to 60%. Violin plots show Pearson correlations across 1,000 random split-half iterations at each density; black bars indicate medians. (B) Observed split-half reliability compared with spatial null models. Observed correlations remained higher than both free-shuffle and lobe-restricted ROI-label permutation null distributions across densities. Within-lobe partial correlations are also shown. (C) Backbone-score profiles across densities, with ROIs ranked by backbone score. Values show highly similar score profiles across thresholds, with narrow bootstrap confidence intervals. (D) Correlation matrix of backbone-score maps across densities, showing strong cross-density similarity. The dashed line marks the primary 30% density threshold used in the main analyses. (E) Per-ROI bootstrap precision across densities, quantified using the bootstrap ratio, defined as the observed backbone score divided by its bootstrap standard deviation. Percentages indicate the proportion of ROIs with (|BSR| > 2). (F) Together, these analyses indicate that the degree-centrality backbone was robust across network density thresholds and was not driven by the specific 30% threshold used in the main figure.

***Supplementary Fig.7.* Relationships among backbone metrics across density thresholds.** Scatter plots show pairwise relationships among mean degree, coefficient of variation (CV), and rank stability across ROIs at network density thresholds from 10% to 60%. Each point represents one ROI and is colored by its continuous backbone score. Red outlines indicate core backbone regions defined by joint criteria: top-quartile mean degree, below-median CV, and rank stability of at least 0.75 across clips. Across densities, mean degree was negatively correlated with CV and positively correlated with rank stability, whereas CV was negatively correlated with rank stability (5,000 permutations; all p_perm = 2 × 10⁻⁴, ***). These results indicate that the three metrics were strongly related but non-equivalent, supporting the use of a composite backbone score and joint criteria to characterize core backbone regions.

***Supplementary Fig. 8. Semantic and human-related stimulus features across movie clips.***

(A) WordNet-based semantic labels across clips. For each movie clip, TR-resolved semantic annotations from the HCP 7T movie-watching dataset were summarized as word clouds, illustrating the most frequently occurring WordNet label categories within each clip. (B) TR-wise human-related features across clips. For each clip, time-resolved traces show the presence and intensity of human-related features across TRs, including the number of detected people (stratified by viewing distance: close/medium/far), face counts, binary indicators of speech and dialogue, and a derived social-strength measure.

***Supplementary Fig.9*. Stimulus structure of movie clips across runs.** (A) Clip-wise semantic profiles derived from TR-resolved annotations provided by the HCP 7T movie-watching dataset. Each movie clip was characterized along 18 predefined semantic dimensions. Values represent the mean feature strength across all TRs within each clip.Heatmap showing z-scored values of 18 predefined semantic dimensions for each movie. (B) Feature-by-feature semantic similarity. Pairwise Pearson correlation matrix between semantic features computed across clips using z-scored feature values. Positive correlations indicate semantic dimensions that tend to co-occur across clips (e.g., speech-related and social features), whereas negative correlations reflect opposing semantic profiles. The diagonal represents self-correlations. (C) Clip-by-clip semantic similarity. Pairwise Pearson correlation matrix between movie clips based on their full semantic feature profiles. Higher similarity values indicate clips with comparable semantic composition, revealing structured relationships both within and across movie runs.

***Supplementary Fig.10.* PCA loading patterns used for stimulus–brain CCA.** PCA was applied separately to the clip-level stimulus-feature matrix and the clip-level node-degree matrix. Bar plots show the loading profiles of the first two stimulus PCs, and brain maps show the loading patterns of the first two brain PCs projected onto cortical and subcortical surfaces.

***Supplementary Fig. 11.*** Full-pipeline permutation and leave-one-clip-out validation of the PCA–CCA analysis across network density thresholds. (A) In-sample canonical correlations were evaluated against full-pipeline permutation null distributions across proportional network density thresholds from 10% to 60%. Grey histograms show permutation null distributions, red dashed lines indicate observed canonical correlations, and black dotted lines indicate the 95th percentile of the null distribution. The observed in-sample canonical correlations exceeded the permutation null at all thresholds. (B) Leave-one-clip-out cross-validation (LOOCV) correlations evaluated against full-pipeline permutation null distributions. LOOCV correlations remained positive across thresholds but did not reach significance. (C) Scatterplots show held-out brain and stimulus canonical scores for each clip across thresholds. Movie 3, clip 4 is circled in red and showed an atypical pattern relative to the other clips. Point color indicates the change in LOOCV correlation when each clip was removed. (D) Left: LOOCV correlations across thresholds with all 14 clips included and after excluding Movie 3, clip 4. Right: per-clip influence, quantified as the mean absolute change in LOOCV correlation across thresholds when each clip was removed. Movie 3, clip 4 had the largest influence on cross-validated performance. These analyses indicate that the in-sample stimulus–brain association was robust across density thresholds, whereas out-of-sample stability was positive but sensitive to individual clips.

**Supplementary Fig. 12. Stimulus feature relevance to the CCA canonical axis.** Scatter plots showing the relationship between the CCA-derived semantic score and individual semantic feature measures across movie clips. Each panel corresponds to one stimuli feature. Each point represents one movie clip. The x-axis denotes the CCA semantic score obtained from canonical correlation analysis, and the y-axis denotes the z-scored value of the corresponding semantic feature. Solid lines indicate least-squares regression fits for visualization. Spearman rank correlation coefficients (r) and associated p values are reported in each panel, quantifying the monotonic relationship between individual semantic features and the CCA stimuli dimension.

***Supplementary Fig. 13.*** **Rich-club coefficient curves for each movie clip at the primary 30% network density threshold.** Rich-club organization was evaluated separately for each movie clip using the group-averaged ISFC matrix. Each subplot shows the rich-club coefficient across a range of degree thresholds (k) for the whole-brain network of one clip. The blue line shows the observed rich-club coefficient, the yellow line shows the mean coefficient from degree-preserving random networks, and the red line shows the normalized rich-club coefficient. Rich-club regions were identified from the significant range of normalized rich-club coefficients, as described in the Methods. This figure illustrates the hub-identification procedure at the primary 30% threshold; threshold sensitivity of rich-club membership across 10%–60% network densities is shown in Supplementary Fig. 14.

***Supplementary Fig. 14.* Threshold sensitivity of rich-club hub organization across movie clips.**

Rich-club regions were identified separately for each movie clip across proportional network density thresholds from 10% to 60%. Red regions indicate ROIs classified as rich-club hubs based on significant normalized rich-club organization relative to degree-preserving null networks. Panels are organized by movie run and clip, with rows showing different network density thresholds. Rich-club membership showed clear threshold dependence, with sparser thresholds yielding more restricted hub sets and denser thresholds producing more spatially extensive rich-club regions in some clips. Despite this variability, rich-club hubs were most consistently observed in posterior temporal, occipital, and parietal regions, supporting a posterior-dominant hub organization during naturalistic movie watching.

***Supplementary Fig. 15*.** **Connector-hub profile of rich-club regions at the primary 30% network density threshold.** (A) Community organization of each movie clip based on the Louvain community detection algorithm. For each clip, brain regions with the same color belong to the same community. The numbers shown in parentheses on the color bar represent the number of brain regions comprising the corresponding community. (B) participation coefficient map of rich-club regions for each clip. Purple: connector hubs (PC > 0.5); green: local hubs (PC ≤ 0.5). (C) Distribution of PC values across clips.

***Supplementary Fig 16.  Whole-brain connectivity profiles of rich-club regions at the primary 30% network density threshold.*** *Rich-club regions are categorized into speech, visual, motor and domain-general systems based on their spatial locations and previously reported primary functions. (A) Categorization and anatomical locations of brain regions. (B) Connections between the rich-club regions and other non-rich-club regions are depicted using circular plots. Red: positive connections; blue: negative connections.*

***Supplementary Fig 17.  Exploratory indirect-association results across stimulus features***. *Forest plots show standardized indirect-association terms ((ab)) for pathways that survived correction for multiple comparisons (p < 0.05, FDR-corrected). Points indicate the mean participant-level indirect term, blue intervals indicate participant-bootstrap 95% confidence intervals, and gray intervals indicate delete-one-clip jackknife 95% confidence intervals. The lower scatter plots provide diagnostic summaries showing the relationship between the indirect term and the stimulus–hub connectivity path, and between the indirect term and stimulus-independent hub–target coupling. Red points indicate pathways surviving the correction threshold. These analyses summarize feature-specific indirect associations among clip-level stimulus features, rich-club hub-to-target connectivity, and within-target-network connectivity.*

***Supplementary table***

| ***Supplementary Table 1***  ***Description of Movie runs. Movie1 and Movie3 consist of clips from independent films available under a Creative Commons license, while Movie2 and Movie4 include clips from Hollywood films.*** | | | | | | | | |
| --- | --- | --- | --- | --- | --- | --- | --- | --- |
| ***Clip number*** | ***Movie1*** | | ***Movie2*** | | ***Movie3*** | | ***Movie4*** | |
|  | ***Leng (TRs)*** | ***Stimuli*** | ***Leng (TRs)*** | ***Stimuli*** | ***Leng (TRs)*** | ***Stimuli*** | ***Leng (TRs)*** | ***Stimuli*** |
|  | ***20*** | *REST* | ***20*** | *REST* | ***20*** | *REST* | ***20*** | *REST* |
| ***Clip1*** | *244* | *Two Men (2009)* | *227* | *Inception (2010)* | *180* | *Off the Shelf: flower (2008)* | *233* | *Home Alone (1990)* |
|  | ***20*** | *REST* | ***20*** | *REST* | ***20*** | *REST* | ***20*** | *REST* |
| ***Clip2*** | *220* | *Welcome to Bridgeville (2011)* | *259* | *The Social Network (2010)* | *184* | *1212: hotel (year unavailable)* | *230* | *Erin Brockovich (2000)* |
|  | ***20*** | *REST* | ***20*** | *REST* | ***20*** | *REST* | ***20*** | *REST* |
| ***Clip3*** | *188* | *Pockets (2008)* | *250* | *Ocean’s Eleven (2001)* | *204* | *Mrs. Meyer’s Clean Day: garden (2013)* | *156* | *The Empire Strikes Back (1980)* |
|  | ***20*** | *REST* | ***20*** | *REST* | ***20*** | *REST* | ***20*** | *REST* |
| ***Clip4*** | *64* | *Inside the Human Body: overcome (2011)* | ***80 TRs*** | *Validation clip : Degrees South (2011); LXIV (2011)* | *142* | ***Clip4：*** *Northwest Passage dreary: (year unavailable)* | ***80*** | *Validation clip: Degrees South (2011); LXIV (2011)* |
|  | ***20*** | *REST* | ***20*** | *REST* | ***20*** | *REST* | ***20*** | *REST* |
| ***Clip5*** | ***80*** | *Validation clip : Degrees South (2011); LXIV (2011)* |  |  | ***80*** | *Validation clip: Degrees South (2011); LXIV (2011)* |  |  |
|  | ***20*** | *REST* |  |  | ***20*** | *REST* |  |  |

| ***Supplementary Table 2***  ***Global efficiency differences of ISFC between clip pairs within each movie, across network thresholds*** | | | | | | | | | | |
| --- | --- | --- | --- | --- | --- | --- | --- | --- | --- | --- |
| **Movie 1** | | | | | | | | | | |
| **Threshold** | **clip_1_vs_clip_2** | **clip_1_vs_clip_3** | **clip_1_vs_clip_4** | **clip_1_vs_clip_5** | **clip_2_vs_clip_3** | **clip_2_vs_clip_4** | **clip_2_vs_clip_5** | **clip_3_vs_clip_4** | **clip_3_vs_clip_5** | **clip_4_vs_clip_5** |
| **10** | 0.022 | 0.035 | 0.000 | 0.002 | 0.013 | 0.023 | 0.025 | 0.035 | 0.037 | 0.002 |
| **20** | 0.023 | 0.026 | 0.012 | 0.013 | 0.003 | 0.035 | 0.036 | 0.038 | 0.039 | 0.001 |
| **30** | 0.025 | 0.026 | 0.010 | 0.014 | 0.001 | 0.036 | 0.040 | 0.037 | 0.040 | 0.004 |
| **40** | 0.026 | 0.026 | 0.012 | 0.015 | 0.000 | 0.038 | 0.041 | 0.038 | 0.041 | 0.003 |
| **50** | 0.026 | 0.026 | 0.013 | 0.015 | 0.001 | 0.039 | 0.041 | 0.039 | 0.041 | 0.002 |
| **60** | 0.026 | 0.026 | 0.014 | 0.015 | 0.001 | 0.040 | 0.041 | 0.039 | 0.041 | 0.002 |
| **70** | 0.026 | 0.026 | 0.014 | 0.015 | 0.001 | 0.040 | 0.041 | 0.039 | 0.041 | 0.002 |
| **80** | 0.026 | 0.026 | 0.014 | 0.015 | 0.001 | 0.040 | 0.041 | 0.039 | 0.041 | 0.002 |
| **90** | 0.026 | 0.026 | 0.014 | 0.015 | 0.001 | 0.040 | 0.041 | 0.039 | 0.041 | 0.002 |
| **100** | 0.026 | 0.026 | 0.014 | 0.015 | 0.001 | 0.040 | 0.041 | 0.039 | 0.041 | 0.002 |
| **Movie 2** | | | | | | | | | | |
| **Threshold ( th% of network density)** | **clip_1_vs_clip_2** | **clip_1_vs_clip_3** | **clip_1_vs_clip_4** | **clip_2_vs_clip_3** | **clip_2_vs_clip_4** | **clip_3_vs_clip_4** |  |  |  |  |
| **10** | 0.004 | 0.004 | 0.028 | 0.007 | 0.024 | 0.032 |  |  |  |  |
| **20** | 0.022 | 0.036 | 0.020 | 0.014 | 0.002 | 0.016 |  |  |  |  |
| **30** | 0.041 | 0.058 | 0.044 | 0.017 | 0.003 | 0.014 |  |  |  |  |
| **40** | 0.050 | 0.068 | 0.054 | 0.018 | 0.005 | 0.013 |  |  |  |  |
| **50** | 0.054 | 0.072 | 0.058 | 0.018 | 0.004 | 0.014 |  |  |  |  |
| **60** | 0.054 | 0.073 | 0.059 | 0.019 | 0.005 | 0.014 |  |  |  |  |
| **70** | 0.054 | 0.073 | 0.059 | 0.019 | 0.005 | 0.014 |  |  |  |  |
| **80** | 0.054 | 0.073 | 0.059 | 0.019 | 0.005 | 0.014 |  |  |  |  |
| **90** | 0.054 | 0.073 | 0.059 | 0.019 | 0.005 | 0.014 |  |  |  |  |
| **100** | 0.054 | 0.073 | 0.059 | 0.019 | 0.005 | 0.014 |  |  |  |  |
| **Movie 3** | | | | | | | | | | |
| **Threshold ( th% of network density)** | **clip_1_vs_clip_2** | **clip_1_vs_clip_3** | **clip_1_vs_clip_4** | **clip_1_vs_clip_5** | **clip_2_vs_clip_3** | **clip_2_vs_clip_4** | **clip_2_vs_clip_5** | **clip_3_vs_clip_4** | **clip_3_vs_clip_5** | **clip_4_vs_clip_5** |
| **10** | 0.021 | 0.011 | 0.046 | 0.012 | 0.010 | 0.067 | 0.008 | 0.057 | 0.002 | 0.058 |
| **20** | 0.025 | 0.017 | 0.049 | 0.019 | 0.008 | 0.074 | 0.006 | 0.066 | 0.001 | 0.067 |
| **30** | 0.027 | 0.018 | 0.046 | 0.019 | 0.009 | 0.074 | 0.009 | 0.065 | 0.000 | 0.065 |
| **40** | 0.028 | 0.018 | 0.047 | 0.018 | 0.010 | 0.075 | 0.010 | 0.065 | 0.000 | 0.065 |
| **50** | 0.027 | 0.018 | 0.048 | 0.018 | 0.010 | 0.075 | 0.010 | 0.065 | 0.000 | 0.065 |
| **60** | 0.027 | 0.018 | 0.048 | 0.018 | 0.009 | 0.075 | 0.009 | 0.066 | 0.000 | 0.066 |
| **70** | 0.028 | 0.018 | 0.048 | 0.018 | 0.010 | 0.075 | 0.010 | 0.066 | 0.000 | 0.066 |
| **80** | 0.028 | 0.018 | 0.048 | 0.018 | 0.010 | 0.075 | 0.010 | 0.066 | 0.000 | 0.066 |
| **90** | 0.028 | 0.018 | 0.048 | 0.018 | 0.010 | 0.075 | 0.010 | 0.066 | 0.000 | 0.066 |
| **100** | 0.028 | 0.018 | 0.048 | 0.018 | 0.010 | 0.075 | 0.010 | 0.066 | 0.000 | 0.066 |
| **Movie 4** | | | | | | | | | | |
| **Threshold ( th% of network density)** | **clip_1_vs_clip_2** | **clip_1_vs_clip_3** | **clip_1_vs_clip_4** | **clip_2_vs_clip_3** | **clip_2_vs_clip_4** | **clip_3_vs_clip_4** |  |  |  |  |
| **10** | 0.002 | 0.002 | 0.008 | 0.004 | 0.006 | 0.010 |  |  |  |  |
| **20** | 0.001 | 0.014 | 0.014 | 0.013 | 0.012 | 0.000 |  |  |  |  |
| **30** | 0.003 | 0.008 | 0.017 | 0.005 | 0.014 | 0.009 |  |  |  |  |
| **40** | 0.006 | 0.010 | 0.020 | 0.004 | 0.014 | 0.010 |  |  |  |  |
| **50** | 0.006 | 0.009 | 0.020 | 0.004 | 0.014 | 0.010 |  |  |  |  |
| **60** | 0.006 | 0.009 | 0.020 | 0.004 | 0.014 | 0.010 |  |  |  |  |
| **70** | 0.006 | 0.009 | 0.020 | 0.003 | 0.014 | 0.011 |  |  |  |  |
| **80** | 0.006 | 0.009 | 0.020 | 0.003 | 0.014 | 0.011 |  |  |  |  |
| **90** | 0.006 | 0.009 | 0.020 | 0.003 | 0.014 | 0.011 |  |  |  |  |
| **100** | 0.006 | 0.009 | 0.020 | 0.003 | 0.014 | 0.011 |  |  |  |  |

| ***Supplementary Table 3***  ***Modularity Q scores between clip pairs within each movie, across network thresholds*** | | | | | | | | | | |
| --- | --- | --- | --- | --- | --- | --- | --- | --- | --- | --- |
| **Movie 1** | | | | | | | | | | |
| **Threshold** | **clip_1_vs_clip_2** | **clip_1_vs_clip_3** | **clip_1_vs_clip_4** | **clip_1_vs_clip_5** | **clip_2_vs_clip_3** | **clip_2_vs_clip_4** | **clip_2_vs_clip_5** | **clip_3_vs_clip_4** | **clip_3_vs_clip_5** | **clip_4_vs_clip_5** |
| **10** | 0.009 | 0.000 | 0.016 | 0.041 | 0.009 | 0.025 | 0.032 | 0.015 | 0.041 | 0.057 |
| **20** | 0.020 | 0.045 | 0.029 | 0.013 | 0.065 | 0.049 | 0.007 | 0.016 | 0.058 | 0.042 |
| **30** | 0.008 | 0.070 | 0.028 | 0.005 | 0.078 | 0.036 | 0.013 | 0.042 | 0.065 | 0.022 |
| **40** | 0.005 | 0.083 | 0.025 | 0.015 | 0.088 | 0.030 | 0.020 | 0.058 | 0.068 | 0.010 |
| **50** | 0.013 | 0.083 | 0.031 | 0.022 | 0.096 | 0.044 | 0.035 | 0.052 | 0.060 | 0.009 |
| **60** | 0.023 | 0.068 | 0.020 | 0.006 | 0.091 | 0.043 | 0.029 | 0.048 | 0.062 | 0.014 |
| **70** | 0.016 | 0.076 | 0.036 | 0.013 | 0.092 | 0.053 | 0.030 | 0.039 | 0.062 | 0.023 |
| **80** | 0.032 | 0.072 | 0.033 | 0.010 | 0.104 | 0.065 | 0.042 | 0.040 | 0.062 | 0.022 |
| **90** | 0.027 | 0.075 | 0.035 | 0.005 | 0.102 | 0.062 | 0.032 | 0.039 | 0.070 | 0.031 |
| **100** | 0.002 | 0.104 | 0.064 | 0.042 | 0.102 | 0.062 | 0.039 | 0.039 | 0.062 | 0.023 |
| **Movie 2** | | | | | | | | | | |
| **Threshold ( th% of network density)** | **clip_1_vs_clip_2** | **clip_1_vs_clip_3** | **clip_1_vs_clip_4** | **clip_2_vs_clip_3** | **clip_2_vs_clip_4** | **clip_3_vs_clip_4** |  |  |  |  |
| **10** | 0.096 | 0.007 | 0.000 | 0.088 | 0.096 | 0.007 |  |  |  |  |
| **20** | 0.040 | 0.060 | 0.059 | 0.020 | 0.018 | 0.002 |  |  |  |  |
| **30** | 0.019 | 0.074 | 0.076 | 0.055 | 0.057 | 0.002 |  |  |  |  |
| **40** | 0.010 | 0.082 | 0.099 | 0.072 | 0.088 | 0.016 |  |  |  |  |
| **50** | 0.014 | 0.113 | 0.114 | 0.099 | 0.100 | 0.001 |  |  |  |  |
| **60** | 0.025 | 0.112 | 0.116 | 0.086 | 0.091 | 0.005 |  |  |  |  |
| **70** | 0.028 | 0.125 | 0.107 | 0.097 | 0.079 | 0.018 |  |  |  |  |
| **80** | 0.023 | 0.108 | 0.102 | 0.085 | 0.079 | 0.006 |  |  |  |  |
| **90** | 0.023 | 0.111 | 0.103 | 0.088 | 0.080 | 0.008 |  |  |  |  |
| **100** | 0.027 | 0.111 | 0.102 | 0.084 | 0.075 | 0.008 |  |  |  |  |
| **Movie 3** | | | | | | | | | | |
| **Threshold ( th% of network density)** | **clip_1_vs_clip_2** | **clip_1_vs_clip_3** | **clip_1_vs_clip_4** | **clip_1_vs_clip_5** | **clip_2_vs_clip_3** | **clip_2_vs_clip_4** | **clip_2_vs_clip_5** | **clip_3_vs_clip_4** | **clip_3_vs_clip_5** | **clip_4_vs_clip_5** |
| **10** | 0.077 | 0.063 | 0.025 | 0.077 | 0.014 | 0.052 | 0.000 | 0.038 | 0.014 | 0.052 |
| **20** | 0.080 | 0.069 | 0.143 | 0.081 | 0.011 | 0.063 | 0.001 | 0.074 | 0.011 | 0.062 |
| **30** | 0.080 | 0.074 | 0.143 | 0.085 | 0.006 | 0.063 | 0.005 | 0.069 | 0.011 | 0.059 |
| **40** | 0.094 | 0.053 | 0.154 | 0.071 | 0.041 | 0.060 | 0.023 | 0.101 | 0.018 | 0.083 |
| **50** | 0.105 | 0.077 | 0.198 | 0.093 | 0.029 | 0.093 | 0.013 | 0.122 | 0.016 | 0.106 |
| **60** | 0.096 | 0.050 | 0.196 | 0.064 | 0.046 | 0.101 | 0.032 | 0.146 | 0.014 | 0.132 |
| **70** | 0.083 | 0.041 | 0.202 | 0.083 | 0.042 | 0.119 | 0.000 | 0.160 | 0.041 | 0.119 |
| **80** | 0.065 | 0.042 | 0.202 | 0.068 | 0.024 | 0.137 | 0.003 | 0.160 | 0.027 | 0.134 |
| **90** | 0.061 | 0.048 | 0.199 | 0.065 | 0.013 | 0.138 | 0.004 | 0.151 | 0.017 | 0.134 |
| **100** | 0.071 | 0.032 | 0.193 | 0.074 | 0.038 | 0.122 | 0.003 | 0.160 | 0.041 | 0.119 |
| **Movie 4** | | | | | | | | | | |
| **Threshold ( th% of network density)** | **clip_1_vs_clip_2** | **clip_1_vs_clip_3** | **clip_1_vs_clip_4** | **clip_2_vs_clip_3** | **clip_2_vs_clip_4** | **clip_3_vs_clip_4** |  |  |  |  |
| **10** | 0.077 | 0.037 | 0.046 | 0.040 | 0.032 | 0.009 |  |  |  |  |
| **20** | 0.033 | 0.038 | 0.052 | 0.005 | 0.019 | 0.013 |  |  |  |  |
| **30** | 0.026 | 0.005 | 0.038 | 0.031 | 0.065 | 0.034 |  |  |  |  |
| **40** | 0.026 | 0.005 | 0.046 | 0.031 | 0.071 | 0.040 |  |  |  |  |
| **50** | 0.027 | 0.003 | 0.055 | 0.030 | 0.082 | 0.052 |  |  |  |  |
| **60** | 0.006 | 0.009 | 0.020 | 0.004 | 0.014 | 0.010 |  |  |  |  |
| **70** | 0.006 | 0.009 | 0.020 | 0.003 | 0.014 | 0.011 |  |  |  |  |
| **80** | 0.006 | 0.009 | 0.020 | 0.003 | 0.014 | 0.011 |  |  |  |  |
| **90** | 0.006 | 0.009 | 0.020 | 0.003 | 0.014 | 0.011 |  |  |  |  |
| **100** | 0.006 | 0.009 | 0.020 | 0.003 | 0.014 | 0.011 |  |  |  |  |

| ***Supplementary Table 4***  *Node-specific metrics of average degree and Coefficient variation* | | | |
| --- | --- | --- | --- |
| **Lobe_name** | **ROI_label** | **Degree valua** | **Coefficient variation** |
| Primary_Visual_L | L_V1 | 154 | 0.31 |
| MT+_Complex_and_Neighboring_Visual_Areas_L | L_MST | 160 | 0.27 |
| Dorsal_Stream_Visual_L | L_V6 | 92 | 0.41 |
| Early_Visual_L | L_V2 | 163 | 0.28 |
| Early_Visual_L | L_V3 | 165 | 0.35 |
| Early_Visual_L | L_V4 | 196 | 0.20 |
| Ventral_Stream_Visual_L | L_V8 | 176 | 0.29 |
| Somatosensory_and_Motor_L | L_4 | 86 | 0.52 |
| Somatosensory_and_Motor_L | L_3b | 82 | 0.52 |
| Premotor_L | L_FEF | 64 | 0.55 |
| Premotor_L | L_PEF | 85 | 0.62 |
| Premotor_L | L_55b | 113 | 0.43 |
| Dorsal_Stream_Visual_L | L_V3A | 146 | 0.32 |
| Posterior_Cingulate_L | L_RSC | 128 | 0.30 |
| Posterior_Cingulate_L | L_POS2 | 145 | 0.31 |
| Dorsal_Stream_Visual_L | L_V7 | 193 | 0.22 |
| Dorsal_Stream_Visual_L | L_IPS1 | 175 | 0.22 |
| Ventral_Stream_Visual_L | L_FFC | 162 | 0.33 |
| Dorsal_Stream_Visual_L | L_V3B | 169 | 0.33 |
| MT+_Complex_and_Neighboring_Visual_Areas_L | L_LO1 | 201 | 0.16 |
| MT+_Complex_and_Neighboring_Visual_Areas_L | L_LO2 | 196 | 0.21 |
| Ventral_Stream_Visual_L | L_PIT | 173 | 0.28 |
| MT+_Complex_and_Neighboring_Visual_Areas_L | L_MT | 172 | 0.26 |
| Early_Auditory_L | L_A1 | 136 | 0.30 |
| Temporo-Parieto-Occipital_Junction_L | L_PSL | 139 | 0.37 |
| Dorsolateral_Prefrontal_L | L_SFL | 137 | 0.32 |
| Posterior_Cingulate_L | L_PCV | 132 | 0.33 |
| Temporo-Parieto-Occipital_Junction_L | L_STV | 157 | 0.25 |
| Superior_Parietal_L | L_7Pm | 138 | 0.39 |
| Posterior_Cingulate_L | L_7m | 145 | 0.22 |
| Posterior_Cingulate_L | L_POS1 | 133 | 0.41 |
| Posterior_Cingulate_L | L_23d | 108 | 0.38 |
| Posterior_Cingulate_L | L_v23ab | 80 | 0.40 |
| Posterior_Cingulate_L | L_d23ab | 125 | 0.34 |
| Posterior_Cingulate_L | L_31pv | 127 | 0.26 |
| Paracentral_Lobular_and_Mid_Cingulate_L | L_5m | 56 | 0.54 |
| Paracentral_Lobular_and_Mid_Cingulate_L | L_5mv | 96 | 0.38 |
| Paracentral_Lobular_and_Mid_Cingulate_L | L_23c | 108 | 0.35 |
| Paracentral_Lobular_and_Mid_Cingulate_L | L_5L | 99 | 0.43 |
| Paracentral_Lobular_and_Mid_Cingulate_L | L_24dd | 62 | 0.51 |
| Paracentral_Lobular_and_Mid_Cingulate_L | L_24dv | 52 | 0.59 |
| Superior_Parietal_L | L_7AL | 148 | 0.33 |
| Paracentral_Lobular_and_Mid_Cingulate_L | L_SCEF | 86 | 0.41 |
| Paracentral_Lobular_and_Mid_Cingulate_L | L_6ma | 91 | 0.62 |
| Superior_Parietal_L | L_7Am | 131 | 0.35 |
| Superior_Parietal_L | L_7PL | 139 | 0.31 |
| Superior_Parietal_L | L_7PC | 149 | 0.36 |
| Superior_Parietal_L | L_LIPv | 155 | 0.27 |
| Superior_Parietal_L | L_VIP | 166 | 0.21 |
| Superior_Parietal_L | L_MIP | 127 | 0.32 |
| Somatosensory_and_Motor_L | L_1 | 91 | 0.44 |
| Somatosensory_and_Motor_L | L_2 | 126 | 0.50 |
| Somatosensory_and_Motor_L | L_3a | 89 | 0.38 |
| Premotor_L | L_6d | 62 | 0.54 |
| Paracentral_Lobular_and_Mid_Cingulate_L | L_6mp | 54 | 0.68 |
| Premotor_L | L_6v | 100 | 0.59 |
| Anterior_Cingulate_and_Medial_Prefrontal_L | L_p24pr | 55 | 0.51 |
| Anterior_Cingulate_and_Medial_Prefrontal_L | L_33pr | 41 | 1.01 |
| Anterior_Cingulate_and_Medial_Prefrontal_L | L_a24pr | 73 | 0.64 |
| Anterior_Cingulate_and_Medial_Prefrontal_L | L_p32pr | 72 | 0.59 |
| Anterior_Cingulate_and_Medial_Prefrontal_L | L_a24 | 56 | 0.58 |
| Anterior_Cingulate_and_Medial_Prefrontal_L | L_d32 | 103 | 0.33 |
| Anterior_Cingulate_and_Medial_Prefrontal_L | L_8BM | 109 | 0.39 |
| Anterior_Cingulate_and_Medial_Prefrontal_L | L_p32 | 62 | 0.54 |
| Anterior_Cingulate_and_Medial_Prefrontal_L | L_10r | 54 | 0.79 |
| Orbital_and_Polar_Frontal_L | L_47m | 50 | 0.61 |
| Dorsolateral_Prefrontal_L | L_8Av | 149 | 0.22 |
| Dorsolateral_Prefrontal_L | L_8Ad | 88 | 0.31 |
| Anterior_Cingulate_and_Medial_Prefrontal_L | L_9m | 112 | 0.33 |
| Dorsolateral_Prefrontal_L | L_8BL | 121 | 0.33 |
| Dorsolateral_Prefrontal_L | L_9p | 107 | 0.30 |
| Orbital_and_Polar_Frontal_L | L_10d | 89 | 0.40 |
| Dorsolateral_Prefrontal_L | L_8C | 88 | 0.51 |
| Inferior_Frontal_L | L_44 | 117 | 0.39 |
| Inferior_Frontal_L | L_45 | 138 | 0.35 |
| Inferior_Frontal_L | L_47l | 121 | 0.39 |
| Inferior_Frontal_L | L_a47r | 94 | 0.48 |
| Premotor_L | L_6r | 115 | 0.43 |
| Inferior_Frontal_L | L_IFJa | 81 | 0.54 |
| Inferior_Frontal_L | L_IFJp | 85 | 0.51 |
| Inferior_Frontal_L | L_IFSp | 112 | 0.41 |
| Inferior_Frontal_L | L_IFSa | 88 | 0.57 |
| Dorsolateral_Prefrontal_L | L_p9-46v | 80 | 0.48 |
| Dorsolateral_Prefrontal_L | L_46 | 111 | 0.45 |
| Dorsolateral_Prefrontal_L | L_a9-46v | 124 | 0.34 |
| Dorsolateral_Prefrontal_L | L_9-46d | 117 | 0.48 |
| Dorsolateral_Prefrontal_L | L_9a | 141 | 0.20 |
| Anterior_Cingulate_and_Medial_Prefrontal_L | L_10v | 81 | 0.45 |
| Orbital_and_Polar_Frontal_L | L_a10p | 107 | 0.36 |
| Orbital_and_Polar_Frontal_L | L_10pp | 61 | 0.55 |
| Orbital_and_Polar_Frontal_L | L_11l | 101 | 0.40 |
| Orbital_and_Polar_Frontal_L | L_13l | 51 | 0.66 |
| Orbital_and_Polar_Frontal_L | L_OFC | 19 | 0.97 |
| Orbital_and_Polar_Frontal_L | L_47s | 93 | 0.39 |
| Superior_Parietal_L | L_LIPd | 88 | 0.46 |
| Premotor_L | L_6a | 136 | 0.37 |
| Dorsolateral_Prefrontal_L | L_i6-8 | 89 | 0.43 |
| Dorsolateral_Prefrontal_L | L_s6-8 | 87 | 0.39 |
| Posterior_Opercular_L | L_43 | 108 | 0.37 |
| Posterior_Opercular_L | L_OP4 | 124 | 0.27 |
| Posterior_Opercular_L | L_OP1 | 99 | 0.41 |
| Posterior_Opercular_L | L_OP2-3 | 81 | 0.38 |
| Early_Auditory_L | L_52 | 85 | 0.34 |
| Early_Auditory_L | L_RI | 107 | 0.33 |
| Early_Auditory_L | L_PFcm | 131 | 0.38 |
| Insular_and_Frontal_Opercular_L | L_PoI2 | 101 | 0.42 |
| Auditory_Association_L | L_TA2 | 137 | 0.37 |
| Insular_and_Frontal_Opercular_L | L_FOP4 | 106 | 0.40 |
| Insular_and_Frontal_Opercular_L | L_MI | 96 | 0.45 |
| Insular_and_Frontal_Opercular_L | L_Pir | 47 | 0.61 |
| Insular_and_Frontal_Opercular_L | L_AVI | 74 | 0.60 |
| Insular_and_Frontal_Opercular_L | L_AAIC | 63 | 0.59 |
| Posterior_Opercular_L | L_FOP1 | 105 | 0.36 |
| Insular_and_Frontal_Opercular_L | L_FOP3 | 83 | 0.39 |
| Insular_and_Frontal_Opercular_L | L_FOP2 | 81 | 0.59 |
| Inferior_Parietal_L | L_PFt | 137 | 0.49 |
| Superior_Parietal_L | L_AIP | 132 | 0.42 |
| Medial_Temporal_L | L_EC | 18 | 1.01 |
| Medial_Temporal_L | L_PreS | 69 | 0.53 |
| Medial_Temporal_L | L_H | 29 | 0.89 |
| Posterior_Cingulate_L | L_ProS | 88 | 0.42 |
| Medial_Temporal_L | L_PeEc | 65 | 0.66 |
| Auditory_Association_L | L_STGa | 163 | 0.31 |
| Early_Auditory_L | L_PBelt | 150 | 0.30 |
| Auditory_Association_L | L_A5 | 190 | 0.24 |
| Medial_Temporal_L | L_PHA1 | 150 | 0.30 |
| Medial_Temporal_L | L_PHA3 | 142 | 0.26 |
| Auditory_Association_L | L_STSda | 178 | 0.23 |
| Auditory_Association_L | L_STSdp | 184 | 0.26 |
| Auditory_Association_L | L_STSvp | 169 | 0.27 |
| Lateral_Temporal_L | L_TGd | 128 | 0.36 |
| Lateral_Temporal_L | L_TE1a | 147 | 0.30 |
| Lateral_Temporal_L | L_TE1p | 124 | 0.31 |
| Lateral_Temporal_L | L_TE2a | 88 | 0.40 |
| Medial_Temporal_L | L_TF | 101 | 0.41 |
| Lateral_Temporal_L | L_TE2p | 99 | 0.50 |
| Lateral_Temporal_L | L_PHT | 117 | 0.36 |
| MT+_Complex_and_Neighboring_Visual_Areas_L | L_PH | 186 | 0.21 |
| Temporo-Parieto-Occipital_Junction_L | L_TPOJ1 | 172 | 0.26 |
| Temporo-Parieto-Occipital_Junction_L | L_TPOJ2 | 149 | 0.25 |
| Temporo-Parieto-Occipital_Junction_L | L_TPOJ3 | 106 | 0.45 |
| Posterior_Cingulate_L | L_DVT | 149 | 0.26 |
| Inferior_Parietal_L | L_PGp | 183 | 0.21 |
| Inferior_Parietal_L | L_IP2 | 125 | 0.41 |
| Inferior_Parietal_L | L_IP1 | 123 | 0.28 |
| Inferior_Parietal_L | L_IP0 | 176 | 0.28 |
| Inferior_Parietal_L | L_PFop | 133 | 0.41 |
| Inferior_Parietal_L | L_PF | 132 | 0.36 |
| Inferior_Parietal_L | L_PFm | 148 | 0.30 |
| Inferior_Parietal_L | L_PGi | 151 | 0.24 |
| Inferior_Parietal_L | L_PGs | 136 | 0.38 |
| Dorsal_Stream_Visual_L | L_V6A | 168 | 0.20 |
| Ventral_Stream_Visual_L | L_VMV1 | 161 | 0.33 |
| Ventral_Stream_Visual_L | L_VMV3 | 168 | 0.29 |
| Medial_Temporal_L | L_PHA2 | 144 | 0.26 |
| MT+_Complex_and_Neighboring_Visual_Areas_L | L_V4t | 186 | 0.27 |
| MT+_Complex_and_Neighboring_Visual_Areas_L | L_FST | 199 | 0.13 |
| MT+_Complex_and_Neighboring_Visual_Areas_L | L_V3CD | 196 | 0.25 |
| MT+_Complex_and_Neighboring_Visual_Areas_L | L_LO3 | 164 | 0.31 |
| Ventral_Stream_Visual_L | L_VMV2 | 172 | 0.27 |
| Posterior_Cingulate_L | L_31pd | 143 | 0.21 |
| Posterior_Cingulate_L | L_31a | 124 | 0.37 |
| Ventral_Stream_Visual_L | L_VVC | 182 | 0.24 |
| Anterior_Cingulate_and_Medial_Prefrontal_L | L_25 | 12 | 1.22 |
| Anterior_Cingulate_and_Medial_Prefrontal_L | L_s32 | 16 | 1.33 |
| Anterior_Cingulate_and_Medial_Prefrontal_L | L_pOFC | 21 | 0.73 |
| Insular_and_Frontal_Opercular_L | L_PoI1 | 83 | 0.42 |
| Insular_and_Frontal_Opercular_L | L_Ig | 68 | 0.46 |
| Insular_and_Frontal_Opercular_L | L_FOP5 | 84 | 0.37 |
| Orbital_and_Polar_Frontal_L | L_p10p | 131 | 0.27 |
| Inferior_Frontal_L | L_p47r | 77 | 0.54 |
| Lateral_Temporal_L | L_TGv | 111 | 0.38 |
| Early_Auditory_L | L_MBelt | 136 | 0.30 |
| Early_Auditory_L | L_LBelt | 145 | 0.29 |
| Auditory_Association_L | L_A4 | 176 | 0.24 |
| Auditory_Association_L | L_STSva | 147 | 0.30 |
| Lateral_Temporal_L | L_TE1m | 118 | 0.29 |
| Insular_and_Frontal_Opercular_L | L_PI | 82 | 0.50 |
| Anterior_Cingulate_and_Medial_Prefrontal_L | L_a32pr | 102 | 0.49 |
| Anterior_Cingulate_and_Medial_Prefrontal_L | L_p24 | 80 | 0.60 |
| Primary_Visual_R | R_V1 | 157 | 0.31 |
| MT+_Complex_and_Neighboring_Visual_Areas_R | R_MST | 182 | 0.26 |
| Dorsal_Stream_Visual_R | R_V6 | 102 | 0.37 |
| Early_Visual_R | R_V2 | 158 | 0.30 |
| Early_Visual_R | R_V3 | 169 | 0.31 |
| Early_Visual_R | R_V4 | 185 | 0.23 |
| Ventral_Stream_Visual_R | R_V8 | 184 | 0.25 |
| Somatosensory_and_Motor_R | R_4 | 87 | 0.54 |
| Somatosensory_and_Motor_R | R_3b | 90 | 0.49 |
| Premotor_R | R_FEF | 120 | 0.38 |
| Premotor_R | R_PEF | 113 | 0.40 |
| Premotor_R | R_55b | 106 | 0.40 |
| Dorsal_Stream_Visual_R | R_V3A | 135 | 0.38 |
| Posterior_Cingulate_R | R_RSC | 134 | 0.29 |
| Posterior_Cingulate_R | R_POS2 | 147 | 0.30 |
| Dorsal_Stream_Visual_R | R_V7 | 190 | 0.17 |
| Dorsal_Stream_Visual_R | R_IPS1 | 167 | 0.21 |
| Ventral_Stream_Visual_R | R_FFC | 157 | 0.34 |
| Dorsal_Stream_Visual_R | R_V3B | 168 | 0.33 |
| MT+_Complex_and_Neighboring_Visual_Areas_R | R_LO1 | 198 | 0.19 |
| MT+_Complex_and_Neighboring_Visual_Areas_R | R_LO2 | 159 | 0.35 |
| Ventral_Stream_Visual_R | R_PIT | 151 | 0.36 |
| MT+_Complex_and_Neighboring_Visual_Areas_R | R_MT | 175 | 0.25 |
| Early_Auditory_R | R_A1 | 121 | 0.32 |
| Temporo-Parieto-Occipital_Junction_R | R_PSL | 130 | 0.33 |
| Dorsolateral_Prefrontal_R | R_SFL | 127 | 0.32 |
| Posterior_Cingulate_R | R_PCV | 136 | 0.37 |
| Temporo-Parieto-Occipital_Junction_R | R_STV | 137 | 0.31 |
| Superior_Parietal_R | R_7Pm | 129 | 0.34 |
| Posterior_Cingulate_R | R_7m | 131 | 0.31 |
| Posterior_Cingulate_R | R_POS1 | 141 | 0.38 |
| Posterior_Cingulate_R | R_23d | 108 | 0.44 |
| Posterior_Cingulate_R | R_v23ab | 94 | 0.43 |
| Posterior_Cingulate_R | R_d23ab | 111 | 0.33 |
| Posterior_Cingulate_R | R_31pv | 119 | 0.28 |
| Paracentral_Lobular_and_Mid_Cingulate_R | R_5m | 57 | 0.70 |
| Paracentral_Lobular_and_Mid_Cingulate_R | R_5mv | 116 | 0.37 |
| Paracentral_Lobular_and_Mid_Cingulate_R | R_23c | 129 | 0.22 |
| Paracentral_Lobular_and_Mid_Cingulate_R | R_5L | 87 | 0.48 |
| Paracentral_Lobular_and_Mid_Cingulate_R | R_24dd | 68 | 0.52 |
| Paracentral_Lobular_and_Mid_Cingulate_R | R_24dv | 53 | 0.59 |
| Superior_Parietal_R | R_7AL | 149 | 0.32 |
| Paracentral_Lobular_and_Mid_Cingulate_R | R_SCEF | 92 | 0.41 |
| Paracentral_Lobular_and_Mid_Cingulate_R | R_6ma | 101 | 0.53 |
| Superior_Parietal_R | R_7Am | 134 | 0.38 |
| Superior_Parietal_R | R_7PL | 136 | 0.29 |
| Superior_Parietal_R | R_7PC | 157 | 0.34 |
| Superior_Parietal_R | R_LIPv | 182 | 0.20 |
| Superior_Parietal_R | R_VIP | 172 | 0.19 |
| Superior_Parietal_R | R_MIP | 139 | 0.32 |
| Somatosensory_and_Motor_R | R_1 | 75 | 0.51 |
| Somatosensory_and_Motor_R | R_2 | 119 | 0.51 |
| Somatosensory_and_Motor_R | R_3a | 83 | 0.48 |
| Premotor_R | R_6d | 69 | 0.68 |
| Paracentral_Lobular_and_Mid_Cingulate_R | R_6mp | 57 | 0.59 |
| Premotor_R | R_6v | 123 | 0.47 |
| Anterior_Cingulate_and_Medial_Prefrontal_R | R_p24pr | 78 | 0.46 |
| Anterior_Cingulate_and_Medial_Prefrontal_R | R_33pr | 58 | 0.66 |
| Anterior_Cingulate_and_Medial_Prefrontal_R | R_a24pr | 88 | 0.53 |
| Anterior_Cingulate_and_Medial_Prefrontal_R | R_p32pr | 79 | 0.62 |
| Anterior_Cingulate_and_Medial_Prefrontal_R | R_a24 | 67 | 0.52 |
| Anterior_Cingulate_and_Medial_Prefrontal_R | R_d32 | 104 | 0.49 |
| Anterior_Cingulate_and_Medial_Prefrontal_R | R_8BM | 84 | 0.58 |
| Anterior_Cingulate_and_Medial_Prefrontal_R | R_p32 | 88 | 0.52 |
| Anterior_Cingulate_and_Medial_Prefrontal_R | R_10r | 40 | 0.82 |
| Orbital_and_Polar_Frontal_R | R_47m | 35 | 0.86 |
| Dorsolateral_Prefrontal_R | R_8Av | 119 | 0.35 |
| Dorsolateral_Prefrontal_R | R_8Ad | 91 | 0.43 |
| Anterior_Cingulate_and_Medial_Prefrontal_R | R_9m | 108 | 0.35 |
| Dorsolateral_Prefrontal_R | R_8BL | 119 | 0.25 |
| Dorsolateral_Prefrontal_R | R_9p | 93 | 0.24 |
| Orbital_and_Polar_Frontal_R | R_10d | 76 | 0.45 |
| Dorsolateral_Prefrontal_R | R_8C | 85 | 0.56 |
| Inferior_Frontal_R | R_44 | 79 | 0.50 |
| Inferior_Frontal_R | R_45 | 115 | 0.29 |
| Inferior_Frontal_R | R_47l | 59 | 0.51 |
| Inferior_Frontal_R | R_a47r | 118 | 0.29 |
| Premotor_R | R_6r | 98 | 0.54 |
| Inferior_Frontal_R | R_IFJa | 68 | 0.51 |
| Inferior_Frontal_R | R_IFJp | 71 | 0.44 |
| Inferior_Frontal_R | R_IFSp | 72 | 0.40 |
| Inferior_Frontal_R | R_IFSa | 70 | 0.47 |
| Dorsolateral_Prefrontal_R | R_p9-46v | 82 | 0.54 |
| Dorsolateral_Prefrontal_R | R_46 | 124 | 0.42 |
| Dorsolateral_Prefrontal_R | R_a9-46v | 115 | 0.39 |
| Dorsolateral_Prefrontal_R | R_9-46d | 113 | 0.46 |
| Dorsolateral_Prefrontal_R | R_9a | 108 | 0.28 |
| Anterior_Cingulate_and_Medial_Prefrontal_R | R_10v | 80 | 0.64 |
| Orbital_and_Polar_Frontal_R | R_a10p | 86 | 0.49 |
| Orbital_and_Polar_Frontal_R | R_10pp | 55 | 0.54 |
| Orbital_and_Polar_Frontal_R | R_11l | 86 | 0.47 |
| Orbital_and_Polar_Frontal_R | R_13l | 35 | 0.81 |
| Orbital_and_Polar_Frontal_R | R_OFC | 11 | 1.25 |
| Orbital_and_Polar_Frontal_R | R_47s | 71 | 0.40 |
| Superior_Parietal_R | R_LIPd | 81 | 0.51 |
| Premotor_R | R_6a | 146 | 0.36 |
| Dorsolateral_Prefrontal_R | R_i6-8 | 119 | 0.43 |
| Dorsolateral_Prefrontal_R | R_s6-8 | 97 | 0.36 |
| Posterior_Opercular_R | R_43 | 109 | 0.34 |
| Posterior_Opercular_R | R_OP4 | 118 | 0.32 |
| Posterior_Opercular_R | R_OP1 | 84 | 0.47 |
| Posterior_Opercular_R | R_OP2-3 | 74 | 0.38 |
| Early_Auditory_R | R_52 | 75 | 0.42 |
| Early_Auditory_R | R_RI | 91 | 0.39 |
| Early_Auditory_R | R_PFcm | 102 | 0.44 |
| Insular_and_Frontal_Opercular_R | R_PoI2 | 93 | 0.43 |
| Auditory_Association_R | R_TA2 | 106 | 0.36 |
| Insular_and_Frontal_Opercular_R | R_FOP4 | 97 | 0.48 |
| Insular_and_Frontal_Opercular_R | R_MI | 88 | 0.49 |
| Insular_and_Frontal_Opercular_R | R_Pir | 44 | 0.65 |
| Insular_and_Frontal_Opercular_R | R_AVI | 60 | 0.75 |
| Insular_and_Frontal_Opercular_R | R_AAIC | 65 | 0.69 |
| Posterior_Opercular_R | R_FOP1 | 105 | 0.30 |
| Insular_and_Frontal_Opercular_R | R_FOP3 | 57 | 0.56 |
| Insular_and_Frontal_Opercular_R | R_FOP2 | 71 | 0.56 |
| Inferior_Parietal_R | R_PFt | 129 | 0.51 |
| Superior_Parietal_R | R_AIP | 134 | 0.42 |
| Medial_Temporal_R | R_EC | 46 | 0.59 |
| Medial_Temporal_R | R_PreS | 87 | 0.48 |
| Medial_Temporal_R | R_H | 22 | 1.05 |
| Posterior_Cingulate_R | R_ProS | 119 | 0.40 |
| Medial_Temporal_R | R_PeEc | 48 | 0.58 |
| Auditory_Association_R | R_STGa | 153 | 0.29 |
| Early_Auditory_R | R_PBelt | 147 | 0.27 |
| Auditory_Association_R | R_A5 | 184 | 0.24 |
| Medial_Temporal_R | R_PHA1 | 155 | 0.32 |
| Medial_Temporal_R | R_PHA3 | 167 | 0.27 |
| Auditory_Association_R | R_STSda | 160 | 0.24 |
| Auditory_Association_R | R_STSdp | 175 | 0.26 |
| Auditory_Association_R | R_STSvp | 158 | 0.26 |
| Lateral_Temporal_R | R_TGd | 125 | 0.29 |
| Lateral_Temporal_R | R_TE1a | 121 | 0.29 |
| Lateral_Temporal_R | R_TE1p | 78 | 0.45 |
| Lateral_Temporal_R | R_TE2a | 55 | 0.49 |
| Medial_Temporal_R | R_TF | 98 | 0.42 |
| Lateral_Temporal_R | R_TE2p | 145 | 0.30 |
| Lateral_Temporal_R | R_PHT | 90 | 0.53 |
| MT+_Complex_and_Neighboring_Visual_Areas_R | R_PH | 186 | 0.24 |
| Temporo-Parieto-Occipital_Junction_R | R_TPOJ1 | 165 | 0.20 |
| Temporo-Parieto-Occipital_Junction_R | R_TPOJ2 | 144 | 0.31 |
| Temporo-Parieto-Occipital_Junction_R | R_TPOJ3 | 118 | 0.49 |
| Posterior_Cingulate_R | R_DVT | 160 | 0.24 |
| Inferior_Parietal_R | R_PGp | 175 | 0.24 |
| Inferior_Parietal_R | R_IP2 | 106 | 0.47 |
| Inferior_Parietal_R | R_IP1 | 125 | 0.35 |
| Inferior_Parietal_R | R_IP0 | 182 | 0.28 |
| Inferior_Parietal_R | R_PFop | 138 | 0.40 |
| Inferior_Parietal_R | R_PF | 136 | 0.35 |
| Inferior_Parietal_R | R_PFm | 136 | 0.28 |
| Inferior_Parietal_R | R_PGi | 143 | 0.25 |
| Inferior_Parietal_R | R_PGs | 156 | 0.27 |
| Dorsal_Stream_Visual_R | R_V6A | 165 | 0.23 |
| Ventral_Stream_Visual_R | R_VMV1 | 124 | 0.37 |
| Ventral_Stream_Visual_R | R_VMV3 | 182 | 0.28 |
| Medial_Temporal_R | R_PHA2 | 171 | 0.27 |
| MT+_Complex_and_Neighboring_Visual_Areas_R | R_V4t | 186 | 0.28 |
| MT+_Complex_and_Neighboring_Visual_Areas_R | R_FST | 192 | 0.14 |
| MT+_Complex_and_Neighboring_Visual_Areas_R | R_V3CD | 192 | 0.27 |
| MT+_Complex_and_Neighboring_Visual_Areas_R | R_LO3 | 183 | 0.25 |
| Ventral_Stream_Visual_R | R_VMV2 | 179 | 0.30 |
| Posterior_Cingulate_R | R_31pd | 136 | 0.25 |
| Posterior_Cingulate_R | R_31a | 138 | 0.29 |
| Ventral_Stream_Visual_R | R_VVC | 185 | 0.25 |
| Anterior_Cingulate_and_Medial_Prefrontal_R | R_25 | 22 | 1.05 |
| Anterior_Cingulate_and_Medial_Prefrontal_R | R_s32 | 15 | 1.43 |
| Anterior_Cingulate_and_Medial_Prefrontal_R | R_pOFC | 12 | 1.51 |
| Insular_and_Frontal_Opercular_R | R_PoI1 | 61 | 0.51 |
| Insular_and_Frontal_Opercular_R | R_Ig | 46 | 0.59 |
| Insular_and_Frontal_Opercular_R | R_FOP5 | 62 | 0.78 |
| Orbital_and_Polar_Frontal_R | R_p10p | 118 | 0.36 |
| Inferior_Frontal_R | R_p47r | 75 | 0.39 |
| Lateral_Temporal_R | R_TGv | 85 | 0.37 |
| Early_Auditory_R | R_MBelt | 130 | 0.27 |
| Early_Auditory_R | R_LBelt | 128 | 0.28 |
| Auditory_Association_R | R_A4 | 167 | 0.26 |
| Auditory_Association_R | R_STSva | 117 | 0.39 |
| Lateral_Temporal_R | R_TE1m | 85 | 0.41 |
| Insular_and_Frontal_Opercular_R | R_PI | 62 | 0.47 |
| Anterior_Cingulate_and_Medial_Prefrontal_R | R_a32pr | 95 | 0.57 |
| Anterior_Cingulate_and_Medial_Prefrontal_R | R_p24 | 87 | 0.60 |
| Subcortical_L | L_Thalam | 63 | 0.55 |
| Subcortical_L | L_Caudate | 57 | 0.57 |
| Subcortical_L | L_Putamen | 36 | 0.85 |
| Subcortical_L | L_Pallidum | 8 | 1.29 |
| Subcortical_L | L_Hipp | 51 | 0.70 |
| Subcortical_L | L_Amygdala | 78 | 0.52 |
| Subcortical_L | L_Accumbens | 24 | 0.83 |
| Subcortical_L | R_Thalam | 48 | 0.64 |
| Subcortical_R | R_Caudate | 52 | 0.60 |
| Subcortical_R | R_Putamen | 27 | 0.95 |
| Subcortical_R | R_Pallidum | 9 | 1.36 |
| Subcortical_R | R_Hipp | 50 | 0.78 |
| Subcortical_R | R_Amygdala | 85 | 0.47 |
| Subcortical_R | R_Accumbens | 17 | 0.81 |

**Reference**

Bullmore, E., Sporns, O., 2012. The economy of brain network organization. Nat. Rev. Neurosci. https://doi.org/10.1038/nrn3214

Martynenko, I., Koldewyn, K., Downing, P.E., 2025. Social brain responses to natural scene images of social interactions. Soc. Cogn. Affect. Neurosci. 20, 1–15. https://doi.org/10.1093/scan/nsaf057

McNamara, Q., De La Vega, A., Yarkoni, T., 2017. Developing a comprehensive framework for multimodal feature extraction. Proceedings of the ACM SIGKDD International Conference on Knowledge Discovery and Data Mining 1567–1574. https://doi.org/10.1145/3097983.3098075

Plaquet, A., Bredin, H., 2023. Powerset multi-class cross entropy loss for neural speaker diarization. Proceedings of the Annual Conference of the International Speech Communication Association, INTERSPEECH 2023-Augus, 3222–3226. https://doi.org/10.21437/Interspeech.2023-205

Redmon, J., Farhadi, A., 2018. YOLOv3: An Incremental Improvement.

Rubin, T.N., Koyejo, O., Gorgolewski, K.J., Jones, M.N., Poldrack, R.A., Yarkoni, T., 2017. Decoding brain activity using a large-scale probabilistic functional-anatomical atlas of human cognition. PLoS Comput. Biol. 13, 1–24. https://doi.org/10.1371/journal.pcbi.1005649

Rubinov, M., Sporns, O., 2010. Complex network measures of brain connectivity: uses and interpretations. Neuroimage 52, 1059–69. https://doi.org/10.1016/j.neuroimage.2009.10.003

van den Heuvel, M.P., Sporns, O., 2011. Rich-club organization of the human connectome. Journal of Neuroscience 31, 15775–15786. https://doi.org/10.1523/JNEUROSCI.3539-11.2011
